# FGF-23 is a biomarker of RV dysfunction and congestion in patients with HFrEF

**DOI:** 10.1038/s41598-023-42558-4

**Published:** 2023-09-25

**Authors:** Jan Benes, Katerina Kroupova, Martin Kotrc, Jiri Petrak, Petr Jarolim, Vendula Novosadova, Josef Kautzner, Vojtech Melenovsky

**Affiliations:** 1 Department of Cardiology, Institute for Clinical and Experimental Medicine-IKEM, Videnska 1958/9, 140 21 Praha 4, Prague, Czech Republic; 2 Third Faculty of Medicine, Charles University, Prague, Czech Republic; 3 BIOCEV, First Faculty of Medicine, Charles University, Vestec, Czech Republic; 4 Department of Pathology, Brigham and Women’s Hospital, Harvard Medical School, Boston, MA USA; 5 Institute of Molecular Genetics, Academy of Sciences of the Czech Republic, Prague, Czech Republic

**Keywords:** Heart failure, Cardiovascular diseases, Proteomics

## Abstract

There is no biomarker reflecting right ventricular dysfunction in HFrEF patients used in clinical practice. We have aimed to look for a circulating marker of RV dysfunction employing a quantitative proteomic strategy. The Olink Proteomics Multiplex panels (Cardiovascular Disease II, III, Cardiometabolic, and Inflammation Target Panels) identified FGF-23 to be the most differentially abundant (more than 2.5-fold) in blood plasma of HF patients with severe RV dysfunction (n = 30) compared to those with preserved RV function (n = 31). A subsequent ELISA-based confirmatory analysis of circulating FGF-23 in a large cohort of patients (n = 344, 72.7% NYHA III/IV, LVEF 22.5%, 54.1% with moderate/severe RV dysfunction), followed by multivariable regression analysis, revealed that the plasma FGF-23 level was most significantly associated with RV dysfunction grade (*p* = 0.0004) and congestion in the systemic circulation (*p* = 0.03), but not with LV-ejection fraction (*p* = 0.69) or estimated glomerular filtration rate (eGFR, *p* = 0.08). FGF-23 was associated with the degree of RV dysfunction in both sub-cohorts (i.e. in patients with and without congestion, *p* < 0.0001). The association between FGF-23 and RV-dysfunction remained significant after the adjustment for BNP (*p* = 0.01). In contrast, when adjusted for BNP, FGF-23 was no longer associated with LV dysfunction (*p* = 0.59). The Cox proportional hazard model revealed that circulating FGF-23 was significantly associated with adverse outcomes even after adjusting for BNP, LVEF, RV dysfunction grade and eGFR. Circulating FGF-23 is thus a biomarker of right ventricular dysfunction in HFrEF patients regardless of congestion status.

## Introduction

Right ventricular dysfunction is a major complicating condition of heart failure (HF) and is associated with poor prognosis^1^. Unfortunately, no reliable biomarker reflecting RV dysfunction is currently available. Established biomarkers used in cardiovascular medicine for diagnostic and prognostic purposes capture distinct pathophysiological mechanisms; cardiac troponins reflect myocardial damage while natriuretic peptides mirror myocardial stress^2^. However, these biomarkers inform on both left ventricular (e.g. acute coronary syndrome) as well as right ventricular (e.g. pulmonary embolism) pathology^3,4^.

Right ventricle is a low-pressure pump that operates in a relatively narrow zone of pressure changes and its dysfunction might be associated with the release of specific proteins that could be detectable in the circulation^5^. Biomarkers reflecting RV dysfunction could mirror HF progression, herald clinical worsening and increased risk of decompensation; such a biomarker could thus assist in tailoring appropriate HF therapy. RV function plays an especially important role in patients undergoing LV-mechanical circulatory support implantation^6^, and a biomarker mirroring RV function could also assist in pre-LVAD or post-LVAD patient management.

The aim of the study was to identify a new circulating biomarker of RV dysfunction. Multiplexing proteomic analysis based on the proximity extension assay (Olink Proteomics, Uppsala, Sweden) with predefined panels of selected proteins was used to screen patient plasma samples. ELISA was used for confirmation in a large cohort of HF patients.

## Methods

### Patients

Patients with stable HFrEF (LVEF < 40%) of at least 6 months duration were enrolled in the study between 2008 and 2011 in a prospectively defined registry. Patients enrolled in the study were those that were electively hospitalized at the Institute for Clinical and Experimental Medicine in Prague for transplant eligibility evaluation or device implantation. These patients were screened and those receiving stable and optimized medical therapy were enrolled. Patients had to have at least 6-month history of HFrEF and had to receive stable medical therapy for at least three months. Subjects with potentially reversible LV dysfunction (planned valve surgery, revascularization, or tachycardia-induced cardiomyopathy) were excluded. Upon study enrollment, patients completed a Minnesota Living with Heart Failure questionnaire (MLHFQ), underwent clinical assessment (physical examination), echocardiography (Vivid-7 and Vivid-9, General Electric, Milwaukee, Wisconsin) and blood sample collection.

Although only stable patients were enrolled, some of them showed signs of congestion in systemic or pulmonary circulation. Systemic congestion was present if patients had any of the following- enlarged jugular veins, lower limb edema, enlarged liver, positive hepatojugular reflux. Congestion in pulmonary circulation was assessed by auscultation or by chest X-ray evaluation by the attending physician. Patients with self-reported congestion were identified as those that responded to question 1 of MLHFQ with > 2 points (during the past month, has your heart problem prevented you from living as you wanted because it caused swelling in your ankles or legs?).

Left ventricular size and function were assessed according to published guidelines^7^. Right ventricular function was assessed semi-quantitatively (normal RV function, mild, moderate and severe RV dysfunction) in an apical 4-chamber view by using tricuspid annular systolic excursion (M-mode TAPSE) and tissue systolic velocity (Sm) with the following cutoffs: RVD0, normal: TAPSE > 20 mm, Sm > 12 cm/s; RVD1, mild impairment: TAPSE 16 to 20 mm, Sm 9 to 12 cm/s; RVD2, moderate: TAPSE 10 to 15 mm, Sm 6 to 9 cm/s; and RVD3, severe: TAPSE < 10 mm, Sm < 6 cm/s. In case of disagreement of criteria, qualitative visual estimation of RV motion in apical 4-chamber was also taken into account. In a subgroup of patients, RV function was assessed quantitatively as fractional area change (FAC)^8^. Patients were prospectively followed for a median of 3.17 years (IQRs 1.04, 8.05) for the occurrence of an adverse outcome that was defined as the combined endpoint of death, urgent heart transplantation, or ventricular assist device implantation. Due to the fact that time to non-urgent transplantation reflects donor availability rather than recipient’s condition, patients who received a non-urgent heart transplant were censored as having no outcome event at the day of transplantation, as reported before^9^. In most cases, the information about the outcome was derived from internal records of the Institute for Clinical and Experimental Medicine (most patients were followed in our hospital). Whenever the information about the outcome was missing, National Institute of Health Information and Statistics was contacted. This institution maintains information about the living status of all Czech citizens.

All research was performed in accordance with relevant guidelines/regulations, the protocol was approved by the Ethics Committee of the Institute for Clinical and Experimental Medicine and the Thomayer University Hospital, and all subjects signed an informed consent.

### Discovery (Olink) cohort

A quantitative RV function assessment with FAC was performed in 122 HFrEF patients that were subsequently divided into quartiles according to the FAC. Subgroups of 31 patients with severe RV dysfunction (1st quartile of FAC) and 30 patients with preserved RV function (4th quartile of FAC) were used for Olink analysis together with 24 age, sex and body mass index—matched controls. Specimens were collected in EDTA–anticoagulated tubes (Vacuette, Greiner Bio-One, Austria), centrifuged at 2200 g for 10 min, collecting plasma which was stored at − 80 °C. Plasma samples were tested by the proximity extension assay using the Cardiovascular Disease II, Cardiovascular Disease III, Cardiometabolic, and Inflammation Target Panels (Olink Proteomics, Uppsala, Sweden, www.olink.com).

### Confirmatory (ELISA) cohort

A subsequent cohort of 344 HF patients was used for ELISA-based verification analysis. FGF-23 levels were measured using the C-terminal human FGF-23 enzyme-linked immunosorbent assay (Immutopics, San Clemente, California). The interassay coefficients of variation were 11.8% at 29.3 relative units (RU)/ml and 5.6% at 285 RU/ml. BNP was measured by chemiluminescent microparticle immunoassay (CV 4.5%; Architect-BNP; Abbott).

### Statistical evaluation

Olink: data were analyzed using R (version 4.1.0). Data were analyzed using packages *ggplot*, *multcomp* and *lmer*. Data were preprocessed according to Olink instructions. Briefly, all Cq values, which were higher than Cq values for negative control were replaced by values for negative control. Data were recalculated into relative quantities and log2 scaled to get data with normal distribution. The linear model was established for group comparison and contrast were used to find the difference between HFrEF patients and controls and between HFrEF patients with severe RV dysfunction and those with preserved RV function. FDR-adjusted p-value was used to determine statistical significance to eliminate multiple comparison error. Pearson correlation coefficient was calculated for FGF-23 abundances determined in Cardiovascular II and Inflammation panels. ELISA-based FGF-23 data analysis was performed using JMP 11 (SAS Institute Inc, Cary, NC). Data in tables and figures are presented as mean ± standard deviation, median with interquartile ranges (IQRs), or frequency (percent) as appropriate. Unpaired t-test or Mann–Whitney test were used to compare continuous variables between groups as appropriate. Cox´s univariable and multivariable model were used to test the effect of analyzed variables on prognosis.

## Results

### Discovery cohort (Olink proteomics multiplex panels)

**Figure 1:**
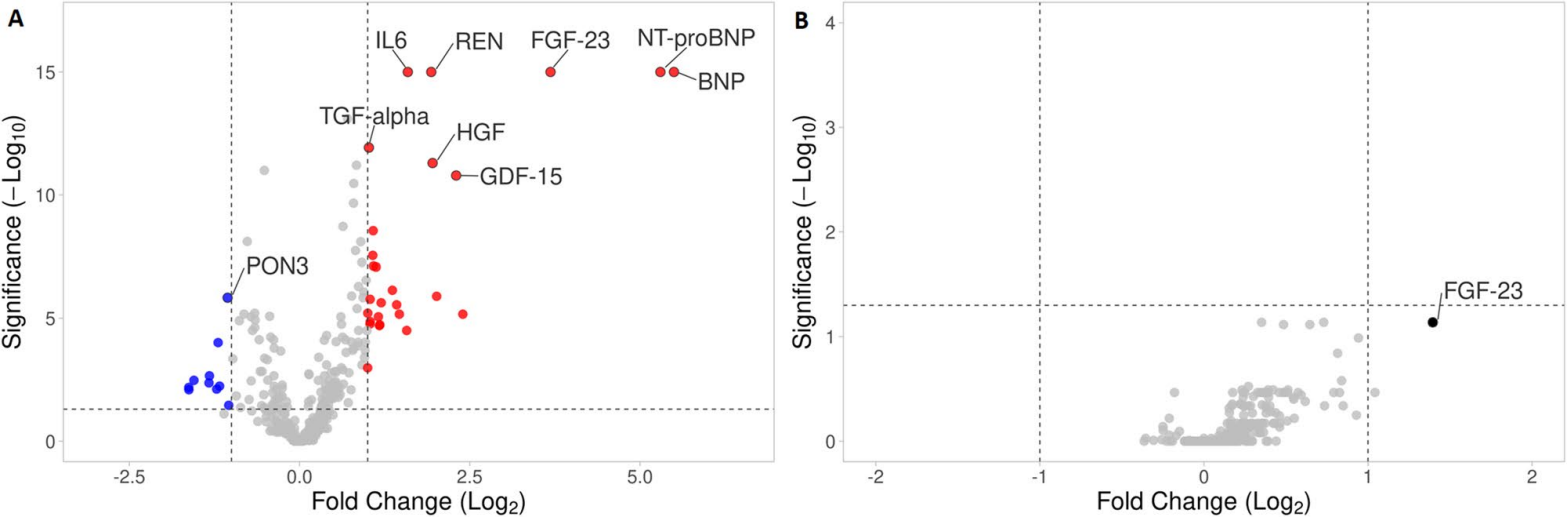
Olink proteomic analysis—Volcano plots showing relative abundances of plasmatic proteins. (**A**) A comparison between HFrEF patients and controls. NT-proBNP, BNP and FGF-23 were among the most markedly and significantly upregulated proteins in HFrEF patients while PON-3 (Serum paraoxonase/lactonase 3) was the most significantly downregulated (**B**) A comparison between HFrEF patients with and without RV dysfunction. FGF-23 was the most markedly upregulated in patients with HFrEF and severe RV dysfunction compared with those with HFrEF and preserved RV function.

In order to identify a circulating biomarker of RV dysfunction we determined relative abundances of 358 proteins in blood plasma samples of HF patients with severe RV dysfunction (n = 31), HF patients with preserved RV function (n = 30) and age/body size matched controls (n = 24) using Olink Target proteomic analysis. Olink Target is a biomarker platform that uses Proximity Extension Assay (PEA) technology combined with qPCR readout to determine relative abundances of selected proteins. Four most relevant Olink panels (Cardiovascular Disease II, Cardiovascular Disease III, Cardiometabolic, and Inflammation Target Panels were used; each of the panel analyzes 92 proteins with a minor overlap of proteins that are included in both panels. Altogether relative abundances of 358 proteins were determined using the four panels. Characteristics of patients and controls is given in Table 1 in the Online Supplement. NT-proBNP, BNP and Fibroblast growth factor-23 (FGF-23) were found to be the most differentially abundant proteins in HF patients compared to controls (Fig. 1A), adjusted *p* < 0.0001. A comparison between patients with preserved RV function and severe RV dysfunction showed FGF-23 to be the most differentially increased protein (> 2.6-fold, adjusted *p* = 0.07, unadjusted *p* = 0.006 Fig. 1B). The complete list of all analyzed proteins is given in Tables 2 and 3 in the Online supplement.

While the selected Olink panels included mostly unique sets of proteins, FGF-23 was captured in both the Cardiovascular II and Inflammation panels, which allowed us to correlate the FGF-23 measurements. Correlation between FGF-23 assessed in both panels was very good (r = 0.99). These results justified a subsequent ELISA-based verification of FGF-23 plasma levels in a larger cohort of patients.

## Confirmatory cohort (ELISA-based FGF-23 analysis)

### Patients

A cohort of 344 patients (84.9% males, 72.7% NYHA III/IV, LVEF 22.5%) with advanced HFrEF were enrolled in the study. A subgroup of 186 patients (54.1%) had moderate or severe RV dysfunction. Patients received a high degree of guideline-directed pharmacotherapy and device therapy—92.7% were treated with beta-blockers, 87.5% with ACEi/ARB and 56.7% with implantable cardioverter-defibrillator (ICD), Table 1. During a follow-up of 3.17 years (IQRs 1.04, 8.05), 247 patients (71.8%) experienced an adverse outcome (death, urgent heart transplantation, mechanical support implantation).

**Table 1:** Patients characteristics.

	Whole cohort (n = 344)	Preserved RV function (n = 72)	Mild RV dysf (n = 86)	Moderate RV dysf (n = 131)	Severe RV dysf (n = 55)	P for trend
Age (years)	57.55 ± 10.36	56.06 ± 10.97	59.41 ± 8.98	57.67 ± 10.93	56.34 ± 9.93	0.96
Males (%)	84.9	70.8	81.4	90.8	94.5	** < 0.0001**
HF etiology (% CAD)	55.8	51.4	53.5	59.8	58.2	0.29
BMI (kg. m^−2^)	27.79 ± 4.77	28.71 ± 4.57	28.48 ± 4.95	26.85 ± 4.48	27.76 ± 5.15	**0.03**
NYHA (2–4,%)	27.3/66.3/6.4	31.9/66.7/1.4	33.7/59.3/7.0	25.2/65.6/9.2	16.4/78.2/5.5	**0.02**
BNP (ng. l^−1^)	568.3 (281.2; 1205.8)	225.9 (116.4; 456.2)	400.4 (228.6; 976.8)	743.6 (415.7; 1366.1)	994.1 (347.7; 1552.7)	** < 0.0001**
SBP (mmHg)	114.58 ± 18.85	121.25 ± 20.70	117.80 ± 19.80	111.03 ± 16.73	109.29 ± 16.37	** < 0.0001**
Hemoglobin (g. l^−1^)	140.57 ± 16.59	136.14 ± 14.60	141.98 ± 17.36	142.43 ± 16.48	139.71 ± 17.37	0.13
eGFR (ml. min^−1^ .1.73 m^−2^)	69.31 ± 21.88	74.44 ± 25.06	67.25 ± 19.86	69.29 ± 21.97	65.87 ± 19.46	0.06
Hb1Ac (mmol/mol)	49.28 ± 15.83	44.35 ± 11.26	48.06 ± 13.66	52.12 ± 19.34	50.93 ± 13.20	**0.002**
Cardiac morphology and function						
LVEDD (mm)	70.94 ± 9.19	69.47 ± 9.38	70.52 ± 9.90	71.22 ± 8.13	72.80 ± 10.03	**0.04**
LVEF (%)	22.52 ± 5.39	25.52 ± 5.66	23.72 ± 5.44	20.99 ± 4.47	20.36 ± 4.77	** < 0.0001**
RVD1 (mm)	39.26 ± 7.85	34.56 ± 6.02	36.40 ± 5.78	40.61 ± 6.36	46.79 ± 9.48	** < 0.0001**
MiR (1–3, %)	9.9/55.5/34.6	12.5/65.3/22.2	9.3/55.8/34.9	7.6/51.1/41.2	12.7/52.7/34.6	0.07
TriR (1–3, %)	32.1/55.3/12.6	58.3/40.3/1.4	37.6/57.7/4.7	23.1/61.5/15.4	9.4/56.6/34.0	** < 0.0001**
IVC (mm)	19.82 ± 5.79	16.74 ± 3.66	18.89 ± 5.00	20.07 ± 5.64	24.64 ± 6.45	** < 0.0001**
Quality of life						
MLHFQ sum	47.77 ± 22.18	42.37 ± 22.81	43.53 ± 21.98	50.75 ± 21.85	53.55 ± 20.45	**0.0007**
MLHFQ somatic	21.66 ± 9.82	19.66 ± 10.19	20.48 ± 9.81	22.56 ± 9.69	23.70 ± 9.27	**0.009**
MLHFQ emotional	8.35 ± 6.26	8.50 ± 6.74	7.38 ± 6.03	8.89 ± 6.14	8.40 ± 6.25	0.58
MLHFQ Q1 (swelling)	1.57 ± 1.88	1.05 ± 1.62	1.00 ± 1.55	1.86 ± 1.99	2.34 ± 1.93	** < 0.0001**
Comorbidities						
Diabetes (%)	162 (47.1)	17 (23.6)	33 (38.4)	78 (59.5)	34 (61.8)	** < 0.0001**
Stroke/TIA (%)	39 (11.3)	8 (11.1)	8 (9.3)	21 (16.0)	2 (3.6)	0.06
COPD (%)	44 (12.8)	10 (13.9)	9 (10.5)	19 (14.5)	6 (10.9)	0.79
Cancer (%)	9 (2.6)	3 (4.2)	1 (1.2)	4 (3.1)	1 (1.8)	0.63
Peripheral arterial disease (%)	35 (10.2)	5 (6.9)	9 (10.5)	16 (12.2)	5 (9.1)	0.67
Therapy						
ACEi/ARB (%)	87.5	88.9	91.9	87.8	78.2	0.13
ACEi/ARB dose (0–3, %)	12.5/48.5/31.1/7.9	11.1/43.1/33.3/12.5	8.1/50/32.6/9.3	12.2/52.6/30.6/4.6	21.8/43.5/27.5/7.2	0.55
BB (%)	92.7	88.9	95.4	92.4	94.6	0.44
BB dose (0–3, %)	7.3/50.2/29.8/12.7	11.1/34.2/38.4/16.3	5.6/47.1/34.7/12.6	7.6/56.4/23.8/12.2	5.4/60.0/25.5/9.1	0.06
MRA (%)	78.5	70.8	79.1	80.9	81.2	0.36
Furosemide daily dose (mg)	80 (40; 125)	60 (40; 80)	60 (40; 125)	80 (40; 125)	120 (60; 125)	** < 0.0001**
ICD any (%)	56.7	61.1	54.7	56.5	54.6	0.84
CRT any (%)	37.8	43.1	29.1	39.7	40	0.26
Outcome						
Death	164 (47.7%)	30 (41.7)	43 (50.0)	66 (50.4)	25 (45.5)	–
Urg. HTx (%)	49 (14.2%)	2 (2.8)	9 (10.5)	23 (17.6)	15 (27.3)	–
Norm. HTx (%)	20 (5.9%)	2 (2.8)	7 (8.1)	9 (6.9)	2 (3.6)	–
MCSi (%)	34 (9.9%)	5 (6.9)	4 (4.7)	15 (11.5)	10 (18.2)	–
Alive with no event	77 (22.4%)	33 (45.8)	23 (26.7)	18 (13.7)	3 (5.5)	–

ACEi, angiotensin-converting enzyme inhibitor; ARB, angiotensin receptor blocker; BB, beta-blocker; BMI, body mass index; BNP, B-type natriuretic peptide; CAD, coronary artery disease; CRT, cardiac resynchronization therapy; eGFR, estimated glomerular filtration rate; Hb1Ac, glycated hemoglobin; HTx, heart transplantation; ICD, implantable cardioverter-defibrillator; IVC, inferior vena cava; LVEDD, left ventricular diameter in diastole; LVEF, left ventricular ejection fraction; MCSi, mechanical circulatory support implantation; MiR, mitral regurgitation; MLHFQ, Minnesota living with heart failure questionnaire; MRA, mineralocorticoid receptor antagonist; NYHA, New York Heart Association; RV, right ventricular; RVD_1_, right ventricle basal diameter in apical four chamber view; TIA, transient ischemic attack; TriR, tricuspid regurgitation.

Beta-blocker and angiotensin-converting enzyme inhibitor/angiotensin receptor blocker dose was evaluated as follows: 0-no dose, 1-low dose (≤ 33% of target dose), 2-intermediate dose (> 33% and ≤ 66% of target dose), and 3-high dose (> 66% of target dose). Significant values are in bold.

### FGF-23 and RV dysfunction

**Figure 2:**
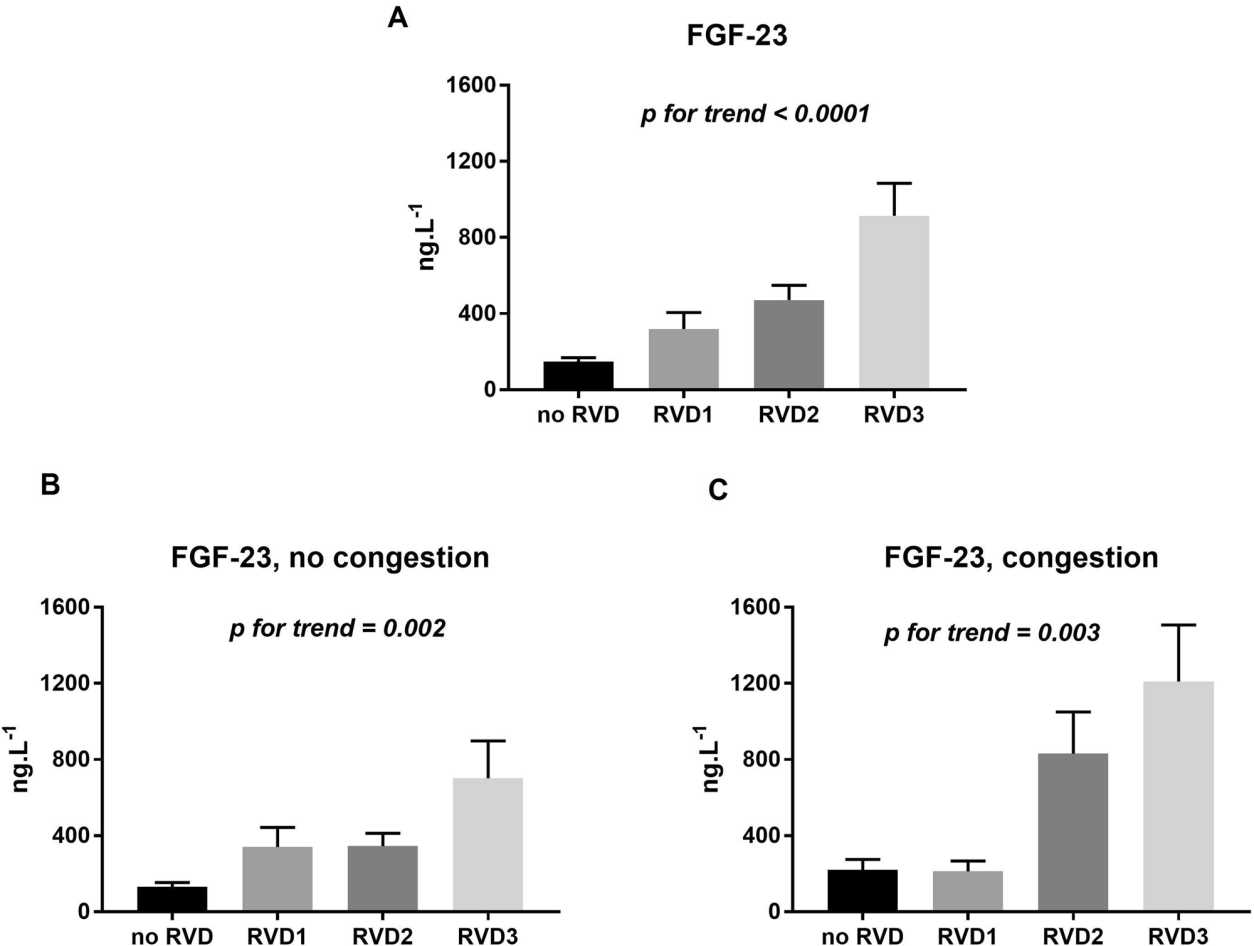
FGF-23 plasma levels determined by ELISA with respect to RV dysfunction grade. (**A**) the whole cohort, (**B**) patients without objective signs of congestion, (**C**) patients with objective signs of congestion Data are presented as mean ± SEM. noRVD—preserved RV function (n = 72), RVD1-mild RV dysfunction (n = 86), RVD2-moderate RV dysfunction (n = 131), RVD3-severe RV dysfunction (n = 55).

Plasmatic FGF-23 concentration determined by ELISA in our cohort ranged from 26.33 to 4451 ng/l with a median of 146.62 ng/l (IQRs 85.64; 352.49). FGF-23 abundance was associated with increasing RV dysfunction grade (Fig. 2A). Regression analysis identified a significant association between FGF-23 and LV-ejection fraction, RV dysfunction grade, congestion (both in the systemic and pulmonary circulation), estimated glomerular filtration rate (eGFR) and glycated hemoglobin level (Hb1Ac, both *p* < 0.05). However, multivariable regression analysis identified that only RV dysfunction grade and congestion in the systemic circulation were most significantly associated with FGF-23 levels (Table 2)**.** In order to investigate the relationship between congestion and RV dysfunction, we have divided the cohort according to the presence or absence of congestion in the systemic circulation (n = 84 and 260, respectively). In both subgroups, FGF-23 significantly increased with increasing RV dysfunction grade (Fig. 2B, C). Since the degree of congestion might vary over time, we investigated the impact of self-reported severity of congestion. Subjective perception of congestion was reported by 98 patients, 217 patients were without the subjective perception of congestion (information was missing in 29 patients). Similarly to patients with or without objective signs of congestion, FGF-23 progressively increased with increasing RV dysfunction grade independently of subjective perception of congestion (*p* for trend = 0.01 and 0.003, respectively, Fig. 1 and Table 4in the online supplement). Thus, plasmatic FGF-23 levels increased with worsening RV function regardless of congestion in the systemic circulation or subjective perception of congestion.

**Table 2:** Parameters associated with FGF-23 level.

Variable	Univariable regression		Multivariable regression
	r^2^	*p*	*p*
LVEF (%)	0.014	**0.03**	0.68
RV dysfunction grade (1–4)	0.07	** < 0.0001**	**0.0004**
Congestion in the pulmonary circulation (present vs. absent)	0.02	**0.008**	0.43
Congestion in the systemic circulation (present vs. absent)	0.04	**0.0003**	**0.03**
eGFR (ml. min^−1^ .1.73 m^−2^)	0.02	**0.02**	0.08
Hb1Ac (mmol/mol)	0.01	**0.04**	0.33

FGF-23, fibroblast growth factor 23; LVEF, left ventricular ejection fraction; RV, right ventricular; eGFR, estimated glomerular filtration rate; Hb1Ac, glycated hemoglobin.

Significant values are in bold.

### BNP and FGF-23 with respect to RV and LV dysfunction

BNP is an established biomarker reflecting myocardial wall stress. As patients with worse RV function had also worse LV function (lower LV-ejection fraction), we tried to clarify the informative role of both proteins (BNP, FGF-23) with respect to LV and RV function. Both BNP and FGF-23 plasma levels significantly increased with worsening of RV function (Table 1, Fig. 2A).

Univariable analysis showed that both BNP and FGF-23 were associated with both left and right ventricular dysfunction (Table 3). When combined together, only BNP but not FGF-23 was associated with the degree of LV dysfunction, but both BNP and FGF-23 were associated with the degree of RV dysfunction. This suggests that BNP level is influenced by the degree of both LV and RV dysfunction, whereas FGF-23 abundance is driven specifically by the degree of RV dysfunction. FGF-23 can thus serve as a biomarker of RV dysfunction.

**Table 3:** The relationship of BNP and FGF-23 with respect to LV and RV dysfunction.

	LV-ejection fraction		RV dysfunction grade	
	Univariable	Multivariable	Univariable	Multivariable
BNP	** < 0.0001**	** < 0.0001**	** < 0.0001**	** < 0.0001**
FGF-23	**0.03**	0.59	** < 0.0001**	**0.01**

Only BNP but not FGF-23 was associated with the degree of LV dysfunction, but both BNP and FGF-23 were associated with the degree of RV dysfunction.

BNP-B-type natriuretic peptide; FGF-23—fibroblast growth factor 23.

Significant values are in bold.

### ROC curve analysis

As both BNP and FGF-23 were associated with RV dysfunction, we have analyzed the potential clinical value of FGF-23 compared to conventionally used BNP for the prediction of severe RV dysfunction. The area under the curve (AUC) for FGF-23 was 0.74 (95%, CI 0.69–0.78), which was marginally higher compared to BNP—0.69 (95% CI 0.64–0.74), *p* = 0.29. Based on ROC curve analysis, a BNP level of 500 ng/L was calculated as the optimal cut-off value for the identification of severe RV dysfunction. The positive predictive value of this cut-off concentration was 25.2% and the negative predictive value (NPV) 94.9%.

**Figure 3:**
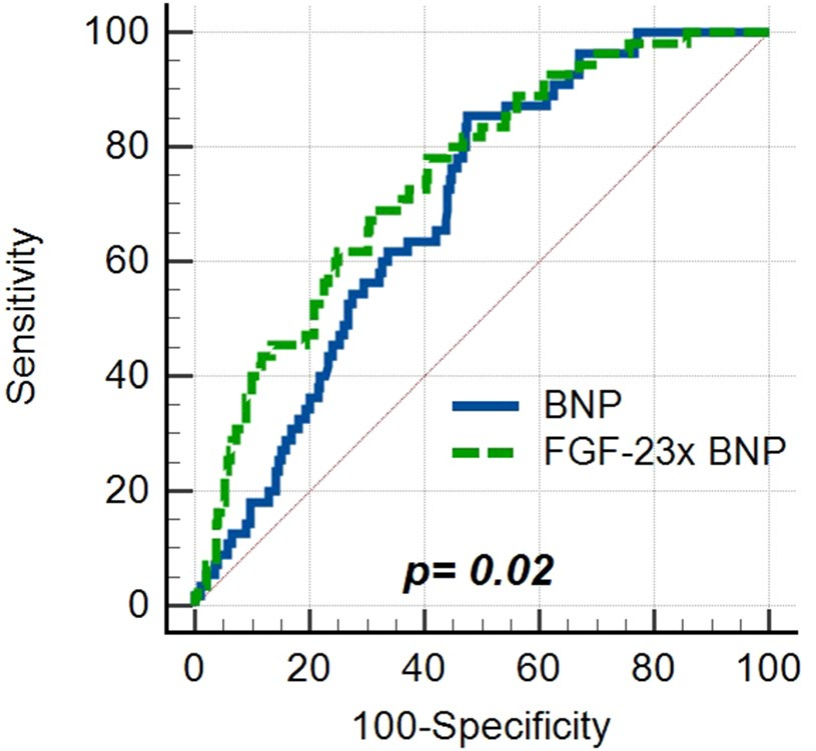
ROC curve analysis. AUC of the numerical product of BNP and FGF-23-0.75 (95%, CI 0.70–0.79) was significantly higher that the AUC of BNP alone − 0.69, (95% CI 0.64–0.74), *p* = 0.02.

The introduction of FGF-23 significantly improved the identification of patients with severe RV dysfunction in our study. AUC of numerical product of BNP and FGF-23 was 0.75 (95%, CI 0.70–0.79), significantly higher that the AUC of BNP alone,* p* = 0.02, Fig. 3. FGF-23 level of 300 ng/L was identified as the optimal cut-off value to distinguish severe RV dysfunction. A combined parameter (BNP > 500 ng/L and FGF-23 > 300 ng/L) improved the positive predictive value for the prediction of severe RV dysfunction. Altogether 42.6% of patients with BNP > 500 ng/L and FGF-23 > 300 ng/L had a severe RV dysfunction; NPV of this combined parameter (91.4%) was comparable to NPV of BNP alone (94.9%).

### Outcome analysis

Finally, the Cox proportional hazard model analysis including major confounders was performed to evaluate the impact of FGF-23 on prognosis of patients.

FGF-23 was significantly associated with adverse outcome even after adjustment for BNP, LV-ejection fraction, RV dysfunction grade and eGFR, Table 4.

**Table 4:** Cox proportional hazard analysis.

Univariable analysis				Multivariable analysis		
	HR	95% CI	*p*	HR	95% CI	*p*
BNP (100 ng/L)	1.06	1.05–1.08	** < 0.0001**	1.04	1.03–1.06	** < 0.0001**
FGF-23 (100 ng/L)	1.04	1.03–1.06	** < 0.0001**	1.02	1.002–1.03	**0.03**
LVEF (5%)	0.80	0.71–0.91	**0.0003**	1.03	0.89–1.18	0.67
RV dysf. grade (1–4)	1.70	1.49–1.94	** < 0.0001**	1.49	1.28–1.73	** < 0.0001**
eGFR (ml. min^−1^ .1.73 m^−2^)	0.99	0.98–0.993	** < 0.0001**	0.99	0.98–0.99	**0.0003**

BNP, B-type natriuretic peptide; FGF-23, fibroblast growth factor 23; LVEF, left ventricular ejection fraction; RV, right ventricular; eGFR, estimated glomerular filtration rate; Hb1Ac, glycated hemoglobin.

Significant values are in bold.

## Discussion

Various biomarkers (troponins, natriuretic peptides)^10^ are used for diagnostic and prognostic purposes in many cardiovascular clinical scenarios (acute coronary syndromes, HF, pulmonary embolism), but currently there is no biomarker specific for RV dysfunction. Proteomic analyses, including the targeted proximity extension-based assay used here, offer a powerful tool for identification of differentially abundant proteins. In our study, fibroblast growth factor-23 (FGF-23) was identified as the strongest upregulated protein in plasma of patients with severe RV dysfunction. Plasmatic FGF-23 level determined by ELISA correlated strongly with the degree of RV dysfunction independent of other possibly confounding variables including subjective perception or objective signs of congestion.

Right ventricle is a low-pressure pump that operates in a relatively narrow zone of pressure changes and its dysfunction might be associated with the release of specific proteins that could be detectable in the circulation^5^. FGF-23 is a 32 kDa secreted protein encoded by the gene *FGF23* located on chromosome 12^11^. FGF-23 acts as endocrine hormone via binding to its receptors FGFR and co-receptor klotho. FGF-23 is involved in phosphate homeostasis; it promotes renal phosphate excretion, decreases the synthesis of 1,25-dihydroxyvitamin D and decreases parathormon (PTH) synthesis^12^. FGF-23 is expressed mainly in osteocytes and osteoblasts in long bones^11^. However, FGF-23 mRNA and protein is also expressed in cardiac myocytes^13–16^. FGF-23 seems to play an important role in cardiac pathophysiology; in vitro it was shown to promote hypertrophy in isolated cardiac myocytes via FGF receptor-dependent activation of calcineurin-NFAT signaling pathway^13,17,18^. In the same study, intramyocardial or intravenous application of FGF-23 resulted in left-ventricular hypertrophy in mice^13^. FGF-23 stimulated proliferation, activation and collagen synthesis in cultured cardiac fibroblasts, while in isolated cardiac myocytes FGF-23 augmented the expression of pro-hypertrophic and pro-inflammatory genes^15^.

Circulating levels of FGF-23 were shown to progressively rise with worsening renal function, which is believed to help to maintain serum phosphate levels in physiological ranges^19^.

Nevertheless, in patients with HF, FGF-23 was not found to be associated with cardiorenal parameters^20^. We have also observed a weak but significant association between eGFR and FGF-23, but this association was no longer significant when adjusted for RV dysfunction. Previous studies have also reported an association between FGF-23 and LV-ejection fraction^21,22^, which was also confirmed in our cohort. Our data suggest that the association between FGF-23 and LVEF is no longer significant when RV dysfunction is taken into account. To our best knowledge, our study is the first study showing the association between FGF-23 and right ventricular dysfunction. Moreover, conventionally used BNP seems to be associated with both LV and RV dysfunction whereas FGF-23 is specific for RV dysfunction only.

Previous studies have documented that plasmatic FGF-23 was also identified as one of the proteins most strongly associated with congestion^23^. It has been shown in experimental settings that peripheral venous congestion was sufficient to cause complex changes in the release of neurohormones and inflammatory mediators^24^. However, this study induced a peripheral congestion by inflating a venous pressure arm cuff. Although peripheral venous congestion mimics notable aspects of the HF congestion phenotype, congestion in the visceral compartment can be further modified by hypoxia and acidosis in enterocytes, increased gut permeability and inflammation and altered renal hemodynamics^24^. We have confirmed and further extended these findings; besides its association with congestion, FGF-23 is related to RV dysfunction regardless of congestion status.

In contrast to FGF-23, BNP (well established marker of HF) was found to reflect the degree of both LV and RV dysfunction. Both LV as well as RV cardiomyocytes are capable of BNP production with a rapid and significant dynamics^10^. Moreover, myocardial stress can reflect not only the degree of RV dysfunction, but RV enlargement as well^25^.

The reasons why plasmatic FGF-23 levels increase in HFrEF patients is unclear and may include multiple mechanisms. Congestion in systemic circulation leads to visceral hypoxia and altered renal hemodynamics, so extra-cardiac production of FGF-23 is conceivable. However, since the association between FGF-23 and RV dysfunction was present even in patients with no overt congestion (or subjective perception of congestion) direct production of FGF-23 by diseased heart is possible as well. Although cardiomyocytes have been shown to be capable of FGF-23 production^14,16,26^, a direct proof that the cardiac tissue or specifically the right ventricle is responsible for FGF-23 production is lacking. This remains to be elucidated by an analysis of fresh or fixed tissue samples or by FGF-23 measurement of blood samples derived from the coronary sinus.

The relationship between FGF-23 level and the degree of RV dysfunction is likely to be non-linear. In patients without congestion, subjects with mild and moderate dysfunction had comparable levels of FGF-23. Similarly, in patients with congestion, subjects with preserved RV function and mild RV dysfunction had comparable levels of FGF-23. Nevertheless, FGF-23 level progressively increased with increasing degree of RV dysfunction grade in both subgroups.

Taken together, since we detected a strong association between FGF-23 and RV dysfunction that is independent of BNP levels and LV-ejection fraction, we propose that FGF-23 may serve as a novel biomarker of RV dysfunction.

### Limitations

Patients were treated not only conservatively (i.e. by optimal pharmacotherapy and ICD/CRT device implantation), but some underwent also heart transplantation or implantation of mechanical circulatory support, which may bias outcome analysis. In addition, it is a single-centre study with a substantial predominance of male patients. Our study cohort included rather young patients with advanced HF but without multiple comorbidities. Consequently, the results might not be fully applicable to patients with milder HF or to older patients. RV dysfunction was assessed semiquantitatively, fractional area change (FAC) as a continuous variable that could be correlated with FGF-23 level was available only in a subgroup of patients. Patients in the confirmatory cohort were enrolled before ARNi and SGLT2i became used for HFrEF treatment. It remains to be investigated whether FGF-23 is a marker of RV dysfunction specific for HFrEF or if it reflects RV dysfunction in other clinical scenarios (HFpEF, pulmonary artery hypertension). The C-terminal human FGF-23 enzyme-linked immunosorbent assay was used in the study. This assay captures both intact (full length) as well as cleaved FGF-23. There are many conditions, however, that impact FGF-23 peptides separately^27^. Therefore, we are not able to distinguish an increased level of FGF-23 due to increased production from increased cleavage. For example iron deficiency, a common finding in HF, upregulates FGF-23 production but also increases cleavege which results in higher levels of c- terminal FGF-23 while the intact form of FGF-23 stays the same.

## Conclusion

Circulating FGF-23 is a biomarker of right ventricular dysfunction in HFrEF patients regardless of congestion status.

### Supplementary Information

Supplementary Figure 1.

Supplementary Table 1.

Supplementary Table 2.

Supplementary Table 3.

Supplementary Table 4.

## Data Availability

The datasets used and/or analysed during the current study are available from the corresponding author on reasonable request.
