# Supplementary figures and images for: FGF-23 is a biomarker of RV dysfunction and congestion in patients with HFrEF
Source: Sci Rep. 2023 Sep 25;13:16004. doi: 10.1038/s41598-023-42558-4 (PMC10520041; doi:10.1038/s41598-023-42558-4)

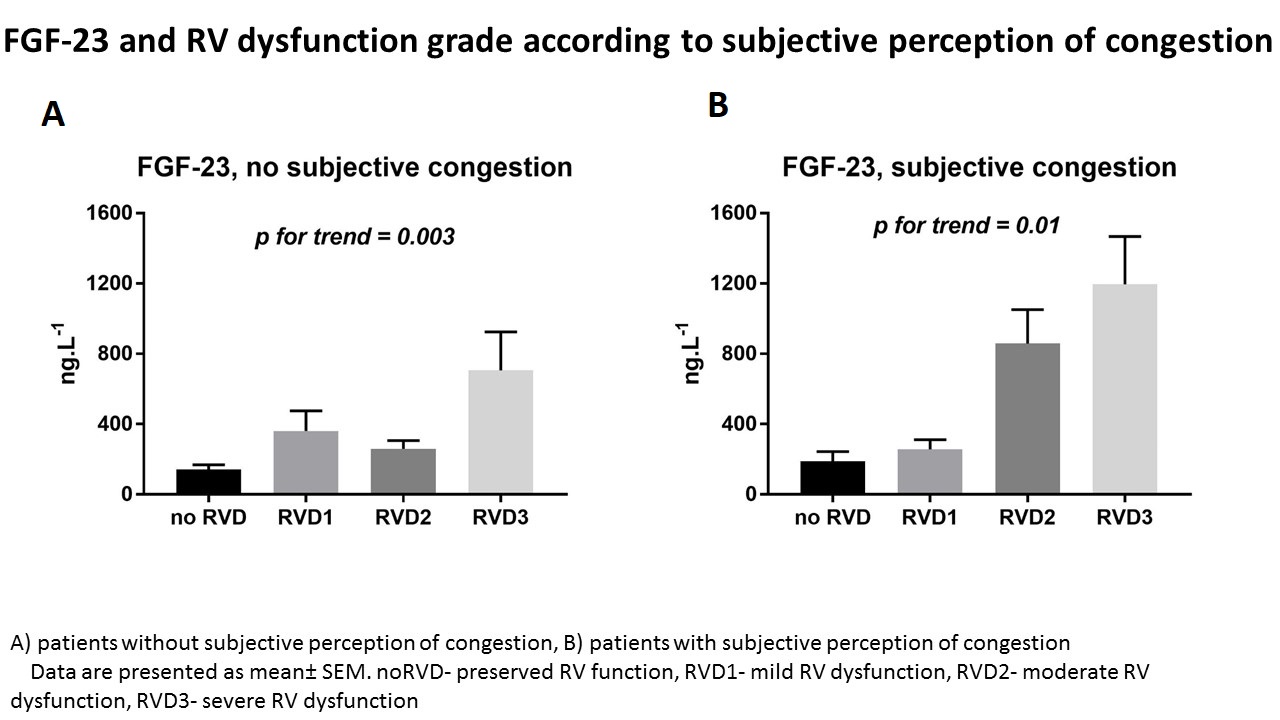

Supplement: Supplementary file 1 — Supplementary Figure 1. [file 41598_2023_42558_MOESM1_ESM.jpg]
