# Supplementary material for: FGF-23 is a biomarker of RV dysfunction and congestion in patients with HFrEF
Source: Sci Rep. 2023 Sep 25;13:16004. doi: 10.1038/s41598-023-42558-4 (PMC10520041; doi:10.1038/s41598-023-42558-4)
Supplement: Supplementary file 2 — Supplementary Table 1. [file 41598_2023_42558_MOESM2_ESM.docx]

**Online supplement Table 1. Characteristics of patients and control used for Olink analysis**

|  | **Controls (n= 24)** | **HFrEF patients (n= 61)** | **HFrEF with severe RV  dysfunction  (n= 31)** | **HFrEF with preserved RV function (n= 30)** | **P** |
| --- | --- | --- | --- | --- | --- |
| Age *(years)* | 51.33 ± 13.50 | 52.25 ± 11.94 | 51.81 ± 11.15 | 52.70 ± 12.89 | 0.77 |
| Males *(%)* | 95.8 | 85.2 | 87.1 | 83.3 | 0.68 |
| HF etiology *(% CAD)* | n/a | 32.8 | 25.8 | 40.0 | 0.23 |
| BMI *(kg.m^-2^)* | 25.62 ± 4.87 | 26.52 ± 3.84 | 26.36± 3.84 | 26.70 ± 3.90 | 0.74 |
| NYHA *(2-4,%)* | n/a | 3.3/ 63.9/ 32.8 | 6.5/ 51.6/ 41.9 | 0/ 76.7/ 23.3 | 0.06 |
| BNP *(ng.l^-1^)* | n/a | 1281 (618; 2549) | 1486 (813; 2927) | 1048 (412; 1973) | 0.09 |
| Creatinine *(µmol.l^-1^)* | n/a | 110.58 ± 40.08 | 106.40 ± 30.21 | 114.89 ± 48.39 | 0.41 |
|  |  |  |  |  |  |
| **Cardiac morphology and function** | | | | | |
| LVEDD *(mm)* | n/a | 73.10 ± 9.28 | 73.77 ± 8.18 | 72.40 ± 10.41 | 0.57 |
| LVEF *(%)* | n/a | 22.09 ± 5.62 | 20.16 ± 2.58 | 24.08 ± 7.09 | **0.005** |
| RVD_1_ *(mm)* | n/a | 45.6± 8.0 | 47.9 ± 5.6 | 43.1 ± 9.3 | **0.02** |
| MiR (1-4, %) | n/a | 18.0/ 29.5/ 27.9/ 24.6 | 19.4/ 35.5/ 25.8/ 19.4 | 16.7/ 23.3/ 30.0/ 30.0 | 0.65 |
| TriR (1-4, %) | n/a | 32.8/ 31.1/ 29.5/ 6.6 | 19.4/ 32.3/ 45.2/ 3.2 | 46.7/ 30.0/ 13.3/ 10.0 | **0.02** |
| FAC | n/a | n/a | 15.4 ± 1.6 | 34.9 ± 5.1 | **< 0.0001** |

BMI, body mass index; CAD, coronary artery disease; LVEDD, left ventricular diameter in diastole; LVEF, left ventricular ejection fraction; MiR, mitral regurgitation; NYHA, New York Heart Association; RV, right ventricular; RVD_1_, right ventricle basal diameter in apical four chamber view; TriR, tricuspid regurgitation
Healthy controls were subjects free of known cardiac disease, renal dysfunction and diabetes (treated arterial hypertension and dyslipidemia were allowed). These subjects have not undergone echocardiography nor laboratory evaluation.
