# Supplementary material for: FGF-23 is a biomarker of RV dysfunction and congestion in patients with HFrEF
Source: Sci Rep. 2023 Sep 25;13:16004. doi: 10.1038/s41598-023-42558-4 (PMC10520041; doi:10.1038/s41598-023-42558-4)
Supplement: Supplementary file 3 — Supplementary Table 2. [file 41598_2023_42558_MOESM3_ESM.pdf]

HFrEF patients versus controls without HF				
Test	gene	Difference (fold-change)	p-value	pvalFDR
Patient vs Ctrl	BNP	5,49832	0,00000	0,00000
Patient vs Ctrl	FGF-23	3,68588	0,00000	0,00000
Patient vs Ctrl	NT-proBNP	5,29970	0,00000	0,00000
Patient vs Ctrl	REN	1,93422	0,00000	0,00000
Patient vs Ctrl	IL6	1,58993	0,00000	0,00000
Patient vs Ctrl	ADM	0,70595	0,00000	0,00000
Patient vs Ctrl	TGF-alpha	1,01821	0,00000	0,00000
Patient vs Ctrl	HGF	1,95385	0,00000	0,00000
Patient vs Ctrl	CD4	0,83619	0,00000	0,00000
Patient vs Ctrl	UMOD	-0,51683	0,00000	0,00000
Patient vs Ctrl	GDF-15	2,29979	0,00000	0,00000
Patient vs Ctrl	CCL3	0,79577	0,00000	0,00000
Patient vs Ctrl	CCL23	0,79241	0,00000	0,00000
Patient vs Ctrl	Gal-9	0,63777	0,00000	0,00000
Patient vs Ctrl	CCL14	1,08130	0,00000	0,00000
Patient vs Ctrl	SCF	-0,76774	0,00000	0,00000
Patient vs Ctrl	PTX3	0,89902	0,00000	0,00000
Patient vs Ctrl	IL-27	0,82243	0,00000	0,00000
Patient vs Ctrl	TRAIL-R2	1,07561	0,00000	0,00000
Patient vs Ctrl	AGRP	0,91464	0,00000	0,00000
Patient vs Ctrl	TNC	1,08646	0,00000	0,00000
Patient vs Ctrl	OSM	1,12479	0,00000	0,00000
Patient vs Ctrl	ACE2	0,97803	0,00000	0,00000
Patient vs Ctrl	CEACAM8	0,86666	0,00000	0,00000
Patient vs Ctrl	IGFBP-2	1,36322	0,00000	0,00000
Patient vs Ctrl	U-PAR	0,94458	0,00000	0,00000
Patient vs Ctrl	CTSL1	0,76544	0,00000	0,00000
Patient vs Ctrl	FABP4	2,01371	0,00000	0,00000
Patient vs Ctrl	COL18A1	0,93294	0,00000	0,00000
Patient vs Ctrl	PON3	-1,05677	0,00000	0,00000
Patient vs Ctrl	TFF3	1,03679	0,00000	0,00000
Patient vs Ctrl	IGFBP-7	1,19891	0,00000	0,00000
Patient vs Ctrl	CCL20	1,42662	0,00000	0,00000
Patient vs Ctrl	MMP-9	0,84438	0,00000	0,00000
Patient vs Ctrl	CCL18	1,00177	0,00000	0,00001
Patient vs Ctrl	APOM	-0,65858	0,00000	0,00001
Patient vs Ctrl	CNDP1	-0,81617	0,00000	0,00001
Patient vs Ctrl	GH	2,39797	0,00000	0,00001
Patient vs Ctrl	FGF-21	1,46577	0,00000	0,00001
Patient vs Ctrl	DNER	-0,43881	0,00000	0,00001
Patient vs Ctrl	CD244	-0,71199	0,00000	0,00001
Patient vs Ctrl	TNFRSF10A	0,60648	0,00000	0,00001
Patient vs Ctrl	IL8	1,15767	0,00000	0,00001
Patient vs Ctrl	PRSS27	-0,65171	0,00000	0,00001
Patient vs Ctrl	TRANCE	-0,88472	0,00000	0,00001
Patient vs Ctrl	OPN	1,03812	0,00000	0,00001
Patient vs Ctrl	vWF	1,03467	0,00000	0,00002
Patient vs Ctrl	LOX-1	0,61611	0,00000	0,00002

Patient vs Ctrl	ST2	1,17950	0,00000	0,00002
Patient vs Ctrl	CXCL9	1,17272	0,00000	0,00002
Patient vs Ctrl	CD84	-0,65736	0,00000	0,00002
Patient vs Ctrl	IGFBP-1	1,57332	0,00000	0,00003
Patient vs Ctrl	NT-3	0,86748	0,00000	0,00003
Patient vs Ctrl	SERPINA5	-0,69086	0,00000	0,00003
Patient vs Ctrl	AZU1	0,98986	0,00000	0,00003
Patient vs Ctrl	OPG	0,39748	0,00001	0,00005
Patient vs Ctrl	TRAIL	-0,42434	0,00001	0,00006
Patient vs Ctrl	PLC	0,63111	0,00001	0,00006
Patient vs Ctrl	F7	-0,59287	0,00001	0,00008
Patient vs Ctrl	HSP 27	-0,38160	0,00001	0,00008
Patient vs Ctrl	CCL15	0,67524	0,00001	0,00008
Patient vs Ctrl	LIF-R	0,36299	0,00001	0,00008
Patient vs Ctrl	TNFRSF13B	0,53685	0,00002	0,00009
Patient vs Ctrl	RETN	0,75736	0,00002	0,00009
Patient vs Ctrl	CCL19	0,86150	0,00002	0,00009
Patient vs Ctrl	TR	0,96516	0,00002	0,00009
Patient vs Ctrl	HB-EGF	-1,19583	0,00002	0,00010
Patient vs Ctrl	SPON1	0,84964	0,00003	0,00013
Patient vs Ctrl	PRCP	-0,37357	0,00003	0,00016
Patient vs Ctrl	C1QTNF1	0,79331	0,00003	0,00017
Patient vs Ctrl	CSTB	0,96226	0,00004	0,00020
Patient vs Ctrl	IL-1ra	0,78175	0,00004	0,00020
Patient vs Ctrl	FAP	-0,27723	0,00004	0,00022
Patient vs Ctrl	CST3	0,68284	0,00005	0,00025
Patient vs Ctrl	PRSS2	0,95353	0,00008	0,00039
Patient vs Ctrl	PRELP	0,28156	0,00008	0,00039
Patient vs Ctrl	CRTAC1	-0,51355	0,00009	0,00042
Patient vs Ctrl	GP6	-0,97940	0,00010	0,00045
Patient vs Ctrl	F11	-0,47362	0,00011	0,00049
Patient vs Ctrl	LTBP2	0,39684	0,00018	0,00079
Patient vs Ctrl	KIM1	0,91677	0,00018	0,00079
Patient vs Ctrl	TNFSF13B	0,61274	0,00024	0,00103
Patient vs Ctrl	CXCL10	1,00003	0,00024	0,00104
Patient vs Ctrl	PGLYRP1	0,63645	0,00029	0,00124
Patient vs Ctrl	TLT-2	-0,57754	0,00035	0,00146
Patient vs Ctrl	MPO	0,51882	0,00035	0,00146
Patient vs Ctrl	THPO	-0,55044	0,00036	0,00146
Patient vs Ctrl	EN-RAGE	0,53573	0,00038	0,00155
Patient vs Ctrl	VSIG2	0,72505	0,00041	0,00167
Patient vs Ctrl	RAGE	0,46260	0,00042	0,00168
Patient vs Ctrl	SPON2	0,13622	0,00047	0,00184
Patient vs Ctrl	PIgR	0,14087	0,00051	0,00199
Patient vs Ctrl	TGM2	0,36676	0,00058	0,00220
Patient vs Ctrl	ST1A1	-1,32345	0,00058	0,00220
Patient vs Ctrl	TM	-0,37652	0,00058	0,00220
Patient vs Ctrl	CD6	-0,46643	0,00089	0,00330
Patient vs Ctrl	AXIN1	-1,55116	0,00091	0,00336
Patient vs Ctrl	DCN	0,27617	0,00092	0,00337

Patient vs Ctrl	CES1	-0,71076	0,00098	0,00355
Patient vs Ctrl	LCN2	0,43019	0,00100	0,00360
Patient vs Ctrl	Gal-4	0,52798	0,00104	0,00368
Patient vs Ctrl	CST5	0,49172	0,00118	0,00416
Patient vs Ctrl	CASP-3	-1,32959	0,00123	0,00428
Patient vs Ctrl	REG1A	0,64440	0,00139	0,00479
Patient vs Ctrl	MCP-3	0,53992	0,00143	0,00487
Patient vs Ctrl	TNF	0,37984	0,00153	0,00515
Patient vs Ctrl	DEFA1	0,46579	0,00154	0,00515
Patient vs Ctrl	PTPRS	-0,25466	0,00158	0,00525
Patient vs Ctrl	TNFRSF11A	0,56142	0,00165	0,00541
Patient vs Ctrl	STK4	-1,17441	0,00178	0,00579
Patient vs Ctrl	SELL	-0,37366	0,00180	0,00581
Patient vs Ctrl	TIMP1	0,50261	0,00189	0,00603
Patient vs Ctrl	MMP-2	0,46974	0,00199	0,00632
Patient vs Ctrl	DECR1	-1,62681	0,00209	0,00655
Patient vs Ctrl	TNF-R1	0,58768	0,00221	0,00687
Patient vs Ctrl	PSGL-1	-0,25474	0,00223	0,00688
Patient vs Ctrl	CXCL5	-1,21671	0,00250	0,00765
Patient vs Ctrl	MEGF9	-0,34010	0,00255	0,00772
Patient vs Ctrl	VEGFD	0,52927	0,00268	0,00801
Patient vs Ctrl	CD59	0,38465	0,00268	0,00801
Patient vs Ctrl	CD40-L	-1,62504	0,00274	0,00810
Patient vs Ctrl	CCL28	0,75644	0,00280	0,00821
Patient vs Ctrl	VCAM1	0,40007	0,00283	0,00824
Patient vs Ctrl	ARTN	0,42812	0,00296	0,00856
Patient vs Ctrl	TIMP4	0,56344	0,00343	0,00984
Patient vs Ctrl	MMP7	0,31490	0,00355	0,01009
Patient vs Ctrl	FCN2	-0,39687	0,00379	0,01068
Patient vs Ctrl	BMP-6	0,44760	0,00405	0,01131
Patient vs Ctrl	PSP-D	0,59677	0,00458	0,01272
Patient vs Ctrl	CTSD	0,49326	0,00474	0,01298
Patient vs Ctrl	PECAM-1	-0,52572	0,00475	0,01298
Patient vs Ctrl	STAMBP	-0,92905	0,00534	0,01447
Patient vs Ctrl	CA4	-0,25462	0,00552	0,01486
Patient vs Ctrl	IL1RL2	-0,28390	0,00556	0,01486
Patient vs Ctrl	CCL16	0,57137	0,00620	0,01645
Patient vs Ctrl	Gal-3	0,38500	0,00654	0,01723
Patient vs Ctrl	GT	0,50559	0,00668	0,01745
Patient vs Ctrl	IGLC2	0,46385	0,00687	0,01782
Patient vs Ctrl	TGFBR3	-0,32103	0,00700	0,01802
Patient vs Ctrl	KIT	-0,30616	0,00712	0,01822
Patient vs Ctrl	ADA	-0,33306	0,00723	0,01835
Patient vs Ctrl	IL-17A	0,47950	0,00738	0,01861
Patient vs Ctrl	NRTN	0,43266	0,00780	0,01949
Patient vs Ctrl	GDNF	0,33422	0,00784	0,01949
Patient vs Ctrl	GIF	-0,74093	0,00825	0,02037
Patient vs Ctrl	THBS2	0,17472	0,00848	0,02078
Patient vs Ctrl	Flt3L	-0,36164	0,00897	0,02185
Patient vs Ctrl	AOC3	0,36981	0,00974	0,02356

Patient vs Ctrl	IL-4RA	0,35469	0,00990	0,02379
Patient vs Ctrl	AMBP	-0,19150	0,01038	0,02479
Patient vs Ctrl	CSF-1	0,13477	0,01048	0,02484
Patient vs Ctrl	CXCL11	0,71543	0,01154	0,02719
Patient vs Ctrl	DPP4	-0,32583	0,01216	0,02845
Patient vs Ctrl	Notch 3	0,37697	0,01272	0,02956
Patient vs Ctrl	FS	0,33174	0,01439	0,03325
Patient vs Ctrl	CCL4	0,42689	0,01452	0,03331
Patient vs Ctrl	PROC	-0,40168	0,01468	0,03347
Patient vs Ctrl	NEMO	-1,03992	0,01535	0,03478
Patient vs Ctrl	CFHR5	-0,38463	0,01560	0,03512
Patient vs Ctrl	MMP-3	-0,55661	0,01612	0,03607
Patient vs Ctrl	GRN	0,32616	0,01656	0,03682
Patient vs Ctrl	CXCL16	0,32453	0,01731	0,03825
Patient vs Ctrl	LIF	0,12933	0,01742	0,03825
Patient vs Ctrl	CD46	-0,39492	0,01847	0,04031
Patient vs Ctrl	MET	-0,17873	0,01930	0,04187
Patient vs Ctrl	TFPI	0,39478	0,01987	0,04260
Patient vs Ctrl	COMP	-0,51745	0,01987	0,04260
Patient vs Ctrl	SIRT2	-0,86645	0,02005	0,04272
Patient vs Ctrl	SELP	-0,52386	0,02090	0,04428
Patient vs Ctrl	PAR-1	-0,31476	0,02117	0,04443
Patient vs Ctrl	CHL1	-0,23539	0,02122	0,04443
Patient vs Ctrl	SERPINA7	-0,30567	0,02317	0,04823
Patient vs Ctrl	CD163	0,41026	0,02395	0,04936
Patient vs Ctrl	ICAM1	0,30820	0,02399	0,04936
Patient vs Ctrl	SOD1	-0,39840	0,02555	0,05226
Patient vs Ctrl	FGF-19	-0,49738	0,02733	0,05540
Patient vs Ctrl	CXCL1	-0,42456	0,02739	0,05540
Patient vs Ctrl	AP-N	0,30862	0,02860	0,05753
Patient vs Ctrl	t-PA	0,44105	0,02975	0,05917
Patient vs Ctrl	SRC	-0,70225	0,02975	0,05917
Patient vs Ctrl	IL-1RT1	0,30357	0,03610	0,07140
Patient vs Ctrl	TNF-R2	0,41630	0,03796	0,07466
Patient vs Ctrl	CDCP1	0,41185	0,03959	0,07744
Patient vs Ctrl	ITGB1BP2	-1,10986	0,03991	0,07766
Patient vs Ctrl	CD5	-0,21482	0,04016	0,07772
Patient vs Ctrl	FETUB	-0,34220	0,04274	0,08226
Patient vs Ctrl	EGFR	-0,22432	0,04321	0,08273
Patient vs Ctrl	CHI3L1	0,49223	0,04509	0,08587
Patient vs Ctrl	IL7	-0,42221	0,04919	0,09318
Patient vs Ctrl	NID1	0,27728	0,05141	0,09687
Patient vs Ctrl	PI3	0,39383	0,05262	0,09863
Patient vs Ctrl	DLK-1	-0,48908	0,05513	0,10280
Patient vs Ctrl	PRTN3	0,32623	0,05750	0,10666
Patient vs Ctrl	MCP-1	0,20000	0,05996	0,11065
Patient vs Ctrl	MMP12	0,40117	0,07055	0,12952
Patient vs Ctrl	TNFRSF9	0,29138	0,07205	0,13160
Patient vs Ctrl	FCGR2A	0,30066	0,07490	0,13558
Patient vs Ctrl	VEGFA	0,25481	0,07525	0,13558

Patient vs Ctrl	PAPPA	0,27203	0,07537	0,13558
Patient vs Ctrl	IgG Fc receptor I	0,35255	0,07930	0,14195
Patient vs Ctrl	IL-12B	0,35176	0,08152	0,14520
Patient vs Ctrl	ADAM-TS13	-0,06996	0,08201	0,14530
Patient vs Ctrl	TNFSF14	-0,35075	0,08239	0,14530
Patient vs Ctrl	IL-18R1	0,21337	0,08519	0,14951
Patient vs Ctrl	CCL5	-0,61234	0,09046	0,15797
Patient vs Ctrl	Dkk-1	-0,34816	0,09439	0,16404
Patient vs Ctrl	JAM-A	-0,48647	0,09513	0,16452
Patient vs Ctrl	TSLP	-0,21990	0,10041	0,17283
Patient vs Ctrl	TR-AP	-0,28844	0,10116	0,17328
Patient vs Ctrl	IL-17D	0,14377	0,10351	0,17590
Patient vs Ctrl	KLK6	0,26439	0,10367	0,17590
Patient vs Ctrl	PLA2G7	-0,16928	0,10426	0,17607
Patient vs Ctrl	NRP1	0,15884	0,10495	0,17639
Patient vs Ctrl	PD-L2	0,20249	0,10605	0,17741
Patient vs Ctrl	GAS6	0,21562	0,10780	0,17950
Patient vs Ctrl	GP1BA	-0,33434	0,10888	0,18014
Patient vs Ctrl	MMP-10	-0,31943	0,10919	0,18014
Patient vs Ctrl	TIMD4	0,28614	0,11327	0,18601
Patient vs Ctrl	NOTCH1	-0,15478	0,12724	0,20800
Patient vs Ctrl	PGF	0,20229	0,13382	0,21686
Patient vs Ctrl	MCP-2	-0,28213	0,13387	0,21686
Patient vs Ctrl	IL5	-0,28166	0,14019	0,22608
Patient vs Ctrl	EFEMP1	0,26458	0,14184	0,22771
Patient vs Ctrl	MFAP5	-0,14752	0,14408	0,23028
Patient vs Ctrl	SLAMF7	0,16458	0,15358	0,24436
Patient vs Ctrl	RARRES2	0,18106	0,16043	0,25413
Patient vs Ctrl	LDL receptor	-0,28416	0,16861	0,26591
Patient vs Ctrl	ENG	0,15578	0,17013	0,26713
Patient vs Ctrl	TNFRSF10C	0,23303	0,17420	0,27124
Patient vs Ctrl	IL2-RA	0,26702	0,17426	0,27124
Patient vs Ctrl	CA1	0,31141	0,17951	0,27821
Patient vs Ctrl	XCL1	0,30725	0,18525	0,28586
Patient vs Ctrl	ANGPTL3	0,20905	0,19302	0,29656
Patient vs Ctrl	IL-2RB	-0,07482	0,19599	0,29881
Patient vs Ctrl	LYVE1	0,21155	0,19689	0,29881
Patient vs Ctrl	CCL24	0,37454	0,19698	0,29881
Patient vs Ctrl	CASP-8	-0,40961	0,19940	0,30120
Patient vs Ctrl	IL10	0,40892	0,20316	0,30486
Patient vs Ctrl	VASN	-0,12474	0,20352	0,30486
Patient vs Ctrl	OSMR	0,13971	0,20475	0,30541
Patient vs Ctrl	SCGB3A2	0,29461	0,20560	0,30541
Patient vs Ctrl	BOC	-0,16437	0,20681	0,30594
Patient vs Ctrl	AXL	0,22506	0,21471	0,31632
Patient vs Ctrl	ANGPT1	-0,33986	0,21919	0,32115
Patient vs Ctrl	IGFBP3	-0,26323	0,21978	0,32115
Patient vs Ctrl	TF	-0,15909	0,22428	0,32639
Patient vs Ctrl	ANG	0,20198	0,22754	0,32980
Patient vs Ctrl	IL-17C	0,28291	0,24875	0,35908

Patient vs Ctrl	IGFBP6	-0,20232	0,25259	0,36316
Patient vs Ctrl	LPL	-0,14650	0,25556	0,36596
Patient vs Ctrl	TNFB	-0,15173	0,25674	0,36619
Patient vs Ctrl	FABP2	0,24372	0,26035	0,36986
Patient vs Ctrl	FAS	-0,19178	0,26716	0,37778
Patient vs Ctrl	PDGF subunit A	-0,31782	0,26874	0,37778
Patient vs Ctrl	PARP-1	0,27924	0,26909	0,37778
Patient vs Ctrl	IL16	-0,19421	0,27107	0,37907
Patient vs Ctrl	CD8A	0,27039	0,27254	0,37965
Patient vs Ctrl	ICAM-2	0,19696	0,27654	0,38225
Patient vs Ctrl	MBL2	-0,43331	0,27655	0,38225
Patient vs Ctrl	CDH1	-0,19259	0,27810	0,38293
Patient vs Ctrl	SERPINA12	-0,39385	0,28080	0,38515
Patient vs Ctrl	CNTN1	-0,16366	0,29531	0,40352
Patient vs Ctrl	IL-20RA	0,23798	0,29975	0,40802
Patient vs Ctrl	CCL25	0,22236	0,30528	0,41398
Patient vs Ctrl	THBS4	-0,29737	0,31674	0,42644
Patient vs Ctrl	FGF-5	0,13220	0,31797	0,42644
Patient vs Ctrl	PDGF subunit B	-0,22956	0,31805	0,42644
Patient vs Ctrl	LTBR	0,18558	0,32193	0,43004
Patient vs Ctrl	Ep-CAM	-0,33178	0,32581	0,43360
Patient vs Ctrl	REG3A	0,09127	0,33949	0,45015
Patient vs Ctrl	4E-BP1	0,18249	0,34185	0,45160
Patient vs Ctrl	SELE	0,20886	0,35837	0,47050
Patient vs Ctrl	LILRB5	0,24076	0,35879	0,47050
Patient vs Ctrl	IL18	-0,11889	0,37514	0,48908
Patient vs Ctrl	IL-17RA	-0,18210	0,37569	0,48908
Patient vs Ctrl	uPA	0,09652	0,37798	0,49028
Patient vs Ctrl	SAA4	-0,20872	0,38262	0,49450
Patient vs Ctrl	PLTP	-0,06803	0,38664	0,49790
Patient vs Ctrl	MERTK	0,12670	0,40413	0,51856
Patient vs Ctrl	IL-15RA	0,13239	0,41884	0,53552
Patient vs Ctrl	hOSCAR	0,06803	0,42336	0,53937
Patient vs Ctrl	ALCAM	0,14432	0,44256	0,56183
Patient vs Ctrl	GDF-2	0,19181	0,45867	0,58022
Patient vs Ctrl	LILRB2	0,13314	0,46622	0,58770
Patient vs Ctrl	CA5A	0,23875	0,46873	0,58880
Patient vs Ctrl	SLAMF1	0,11784	0,49101	0,61122
Patient vs Ctrl	IL-10RB	-0,09565	0,49154	0,61122
Patient vs Ctrl	CD93	0,13618	0,49171	0,61122
Patient vs Ctrl	ST6GAL1	0,12038	0,49954	0,61880
Patient vs Ctrl	IL-20	-0,06603	0,50413	0,62234
Patient vs Ctrl	FCGR3B	0,15313	0,51919	0,63873
Patient vs Ctrl	IL-18BP	0,15201	0,52471	0,64331
Patient vs Ctrl	CPB1	0,20077	0,53654	0,65557
Patient vs Ctrl	PCSK9	0,11582	0,54264	0,66077
Patient vs Ctrl	MB	0,19196	0,56689	0,68796
Patient vs Ctrl	CD40	-0,13872	0,57876	0,69998
Patient vs Ctrl	SPARCL1	-0,08827	0,58757	0,70825
Patient vs Ctrl	TIE1	0,08123	0,59540	0,71528

Patient vs Ctrl	TIE2	0,06143	0,62162	0,74428
Patient vs Ctrl	TCN2	-0,07684	0,62611	0,74716
Patient vs Ctrl	PD-L1	-0,10369	0,64556	0,76781
Patient vs Ctrl	CX3CL1	0,10043	0,64885	0,76916
Patient vs Ctrl	IL7R	0,12082	0,65625	0,77537
Patient vs Ctrl	TNXB	-0,06692	0,67347	0,79310
Patient vs Ctrl	EPHB4	0,10024	0,67604	0,79351
Patient vs Ctrl	GLO1	0,13233	0,68142	0,79657
Patient vs Ctrl	ICAM3	-0,06982	0,68309	0,79657
Patient vs Ctrl	ITGB2	-0,09400	0,68649	0,79793
Patient vs Ctrl	IL-6RA	0,09996	0,69584	0,80618
Patient vs Ctrl	IL-1 alpha	0,13236	0,70362	0,81257
Patient vs Ctrl	IFN-gamma	0,19785	0,73711	0,84599
Patient vs Ctrl	IL-1RT2	0,08763	0,73728	0,84599
Patient vs Ctrl	LAP TGF-beta-1	-0,10076	0,75555	0,86417
Patient vs Ctrl	MCP-4	0,11854	0,75987	0,86635
Patient vs Ctrl	IL4	-0,05162	0,76592	0,87047
Patient vs Ctrl	IL-22 RA1	-0,09926	0,77638	0,87957
Patient vs Ctrl	Beta-NGF	0,00013	0,77910	0,87987
Patient vs Ctrl	IL-10RA	0,07860	0,78606	0,88480
Patient vs Ctrl	SORT1	-0,03995	0,78841	0,88480
Patient vs Ctrl	PAM	-0,05592	0,79301	0,88719
Patient vs Ctrl	TNFRSF14	0,08753	0,80748	0,90055
Patient vs Ctrl	CPA1	0,13208	0,81325	0,90417
Patient vs Ctrl	CDH5	0,07703	0,82075	0,90968
Patient vs Ctrl	CHIT1	0,20201	0,83467	0,92225
Patient vs Ctrl	IL33	-0,09536	0,84645	0,93240
Patient vs Ctrl	CCL17	0,14440	0,85972	0,94411
Patient vs Ctrl	IL13	-0,07746	0,87031	0,95281
Patient vs Ctrl	ITGAM	0,03540	0,87685	0,95704
Patient vs Ctrl	SHPS-1	0,05809	0,88404	0,96197
Patient vs Ctrl	CA3	0,04957	0,88865	0,96405
Patient vs Ctrl	HO-1	-0,03138	0,89658	0,96972
Patient vs Ctrl	PCOLCE	-0,05425	0,90731	0,97631
Patient vs Ctrl	IDUA	-0,04545	0,90813	0,97631
Patient vs Ctrl	PLXNB2	0,03011	0,91891	0,98494
Patient vs Ctrl	SOD2	-0,00868	0,92709	0,98944
Patient vs Ctrl	IL2	0,00959	0,93321	0,98944
Patient vs Ctrl	TWEAK	0,06527	0,93372	0,98944
Patient vs Ctrl	PRSS8	0,02758	0,93417	0,98944
Patient vs Ctrl	LEP	-0,07597	0,94661	0,99947
Patient vs Ctrl	CTRC	-0,06593	0,96430	0,99947
Patient vs Ctrl	TGFBI	-0,02830	0,97036	0,99947
Patient vs Ctrl	GNLY	0,02427	0,97139	0,99947
Patient vs Ctrl	CR2	-0,03314	0,97218	0,99947
Patient vs Ctrl	HAOX1	-0,07349	0,97718	0,99947
Patient vs Ctrl	BLM hydrolase	-0,02414	0,97835	0,99947
Patient vs Ctrl	IL-24	0,01784	0,98026	0,99947
Patient vs Ctrl	CTSZ	-0,01997	0,98239	0,99947
Patient vs Ctrl	LILRB1	0,01366	0,98311	0,99947

Patient vs Ctrl	NCAM1	-0,01748	0,98575	0,99947
Patient vs Ctrl	MEPE	0,02294	0,98801	0,99947
Patient vs Ctrl	C2	-0,00830	0,99534	0,99947
Patient vs Ctrl	CCL11	-0,00731	0,99681	0,99947
Patient vs Ctrl	MARCO	-0,00221	0,99874	0,99947
Patient vs Ctrl	MMP-1	0,01113	0,99878	0,99947
Patient vs Ctrl	PAI	-0,00883	0,99892	0,99947
Patient vs Ctrl	CXCL6	0,00727	0,99921	0,99947
Patient vs Ctrl	QPCT	-0,00321	0,99934	0,99947
Patient vs Ctrl	COL1A1	0,00329	0,99947	0,99947
