# Supplementary material for: FGF-23 is a biomarker of RV dysfunction and congestion in patients with HFrEF
Source: Sci Rep. 2023 Sep 25;13:16004. doi: 10.1038/s41598-023-42558-4 (PMC10520041; doi:10.1038/s41598-023-42558-4)
Supplement: Supplementary file 4 — Supplementary Table 3. [file 41598_2023_42558_MOESM4_ESM.pdf]

HFrEF patients with severe RV dysfunction versus HFrEF patients with preserved RV function				
Test	gene	Difference (fold change)	p-value	pvalFDR
RVD vs. noRVD	FGF-23	1,39507	0,00057	0,07293
RVD vs. noRVD	CXCL5	1,04239	0,01891	0,34238
RVD vs. noRVD	CXCL11	0,94177	0,00172	0,10290
RVD vs. noRVD	GH	0,92912	0,09769	0,56410
RVD vs. noRVD	NT-proBNP	0,84876	0,05757	0,45877
RVD vs. noRVD	MMP-1	0,83952	0,00590	0,26382
RVD vs. noRVD	CCL5	0,82651	0,02636	0,34238
RVD vs. noRVD	CCL28	0,81512	0,00282	0,14424
RVD vs. noRVD	CCL17	0,79173	0,02971	0,34238
RVD vs. noRVD	IGFBP-1	0,73502	0,06006	0,45877
RVD vs. noRVD	MCP-4	0,73005	0,00061	0,07293
RVD vs. noRVD	IL6	0,64592	0,00107	0,07666
RVD vs. noRVD	CCL20	0,61732	0,04540	0,41679
RVD vs. noRVD	CXCL9	0,59269	0,03340	0,36232
RVD vs. noRVD	IGFBP-7	0,57440	0,02263	0,34238
RVD vs. noRVD	GDF-15	0,55049	0,10969	0,60412
RVD vs. noRVD	HGF	0,54741	0,04209	0,39656
RVD vs. noRVD	IL8	0,54488	0,03924	0,37971
RVD vs. noRVD	CXCL1	0,51203	0,01174	0,32327
RVD vs. noRVD	NT-3	0,50793	0,01434	0,34219
RVD vs. noRVD	CXCL10	0,50754	0,11796	0,63982
RVD vs. noRVD	REG1A	0,49202	0,02894	0,34238
RVD vs. noRVD	AP-N	0,48520	0,00094	0,07666
RVD vs. noRVD	CCL18	0,48295	0,02917	0,34238
RVD vs. noRVD	JAM-A	0,46131	0,15768	0,67470
RVD vs. noRVD	BNP	0,46099	0,19865	0,73225
RVD vs. noRVD	PDGF subunit A	0,46013	0,10063	0,57186
RVD vs. noRVD	TNC	0,45965	0,02013	0,34238
RVD vs. noRVD	ITGB1BP2	0,43968	0,62272	1,00000
RVD vs. noRVD	TWEAK	0,43714	0,08662	0,53467
RVD vs. noRVD	ANGPT1	0,43020	0,12796	0,67370
RVD vs. noRVD	THBS4	0,42320	0,14210	0,67470
RVD vs. noRVD	MMP-2	0,42197	0,01122	0,32327
RVD vs. noRVD	SPON1	0,40785	0,08189	0,52351
RVD vs. noRVD	C1QTNF1	0,40160	0,06802	0,45949
RVD vs. noRVD	CST3	0,39361	0,03835	0,37971
RVD vs. noRVD	IL-1RT2	0,39121	0,01008	0,32327
RVD vs. noRVD	CASP-3	0,39072	0,55608	1,00000
RVD vs. noRVD	CCL14	0,39027	0,02741	0,34238
RVD vs. noRVD	FCGR2A	0,38724	0,02747	0,34238
RVD vs. noRVD	GT	0,38095	0,07395	0,48394
RVD vs. noRVD	Dkk-1	0,37932	0,09020	0,53818
RVD vs. noRVD	STK4	0,37884	0,51612	1,00000
RVD vs. noRVD	IL-1RT1	0,37680	0,01371	0,34219
RVD vs. noRVD	COL18A1	0,37380	0,05835	0,45877
RVD vs. noRVD	OPN	0,37256	0,15416	0,67470
RVD vs. noRVD	ST2	0,36384	0,25731	0,82246
RVD vs. noRVD	AOC3	0,36183	0,02055	0,34238

RVD vs. noRVD	IL7	0,35865	0,14522	0,67470
RVD vs. noRVD	t-PA	0,35485	0,12989	0,67393
RVD vs. noRVD	TIMP1	0,35472	0,05402	0,44977
RVD vs. noRVD	OPG	0,35147	0,00031	0,07293
RVD vs. noRVD	TGFBI	0,35117	0,02831	0,34238
RVD vs. noRVD	IGFBP-2	0,34322	0,28412	0,83374
RVD vs. noRVD	ANGPTL3	0,34303	0,02739	0,34238
RVD vs. noRVD	PAI	0,34128	0,26653	0,82358
RVD vs. noRVD	SELP	0,34048	0,22210	0,78726
RVD vs. noRVD	CCL16	0,33411	0,19359	0,73225
RVD vs. noRVD	IgG Fc receptor II-b	0,33340	0,13785	0,67470
RVD vs. noRVD	NID1	0,32643	0,03060	0,34238
RVD vs. noRVD	CXCL6	0,32596	0,26916	0,82358
RVD vs. noRVD	PDGF subunit B	0,31461	0,16353	0,68076
RVD vs. noRVD	CNTN1	0,30161	0,03538	0,37253
RVD vs. noRVD	DECR1	0,29562	0,81286	1,00000
RVD vs. noRVD	AGRP	0,29478	0,08865	0,53793
RVD vs. noRVD	TNFSF13B	0,29015	0,15245	0,67470
RVD vs. noRVD	CTRC	0,28892	0,55514	1,00000
RVD vs. noRVD	LAP TGF-beta-1	0,28850	0,15356	0,67470
RVD vs. noRVD	CCL25	0,28747	0,18672	0,72659
RVD vs. noRVD	TRAIL-R2	0,28537	0,17310	0,71228
RVD vs. noRVD	ICAM1	0,28426	0,06087	0,45877
RVD vs. noRVD	COMP	0,28110	0,33920	0,88536
RVD vs. noRVD	CTSD	0,27868	0,19689	0,73225
RVD vs. noRVD	TFPI	0,27187	0,18218	0,72659
RVD vs. noRVD	TNXB	0,27103	0,00753	0,29947
RVD vs. noRVD	DPP4	0,27021	0,06537	0,45949
RVD vs. noRVD	AXIN1	0,26869	0,80472	1,00000
RVD vs. noRVD	CCL24	0,26588	0,48420	0,99623
RVD vs. noRVD	SRC	0,26386	0,62577	1,00000
RVD vs. noRVD	FCGR3B	0,25670	0,21483	0,77686
RVD vs. noRVD	CD244	0,25654	0,13850	0,67470
RVD vs. noRVD	ANG	0,25245	0,14105	0,67470
RVD vs. noRVD	CCL3	0,25173	0,06786	0,45949
RVD vs. noRVD	IL-1 alpha	0,25099	0,34626	0,88536
RVD vs. noRVD	GP6	0,24912	0,52932	1,00000
RVD vs. noRVD	CHL1	0,24911	0,02417	0,34238
RVD vs. noRVD	TFF3	0,24840	0,34744	0,88536
RVD vs. noRVD	PECAM-1	0,24646	0,32100	0,87935
RVD vs. noRVD	LYVE1	0,24494	0,15578	0,67470
RVD vs. noRVD	IGFBP3	0,24333	0,32385	0,87935
RVD vs. noRVD	EFEMP1	0,24297	0,23707	0,80067
RVD vs. noRVD	Notch 3	0,24277	0,18659	0,72659
RVD vs. noRVD	GDNF	0,24235	0,09597	0,56325
RVD vs. noRVD	KIT	0,24108	0,06151	0,45877
RVD vs. noRVD	TIMD4	0,24095	0,25604	0,82246
RVD vs. noRVD	RARRES2	0,23977	0,06796	0,45949
RVD vs. noRVD	OSMR	0,23964	0,02314	0,34238
RVD vs. noRVD	CD4	0,23925	0,03893	0,37971

RVD vs. noRVD	VCAM1	0,23921	0,13595	0,67470
RVD vs. noRVD	THPO	0,23475	0,23234	0,80067
RVD vs. noRVD	CA1	0,23344	0,42584	0,94146
RVD vs. noRVD	LIF-R	0,23247	0,01088	0,32327
RVD vs. noRVD	IL-27	0,23104	0,13331	0,67470
RVD vs. noRVD	SCGB3A2	0,22990	0,42803	0,94146
RVD vs. noRVD	IL7R	0,22853	0,28387	0,83374
RVD vs. noRVD	PAR-1	0,22783	0,15887	0,67470
RVD vs. noRVD	MCP-1	0,22336	0,04939	0,44127
RVD vs. noRVD	CD40	0,22184	0,30819	0,87771
RVD vs. noRVD	ENG	0,22110	0,05177	0,44127
RVD vs. noRVD	CCL4	0,21682	0,35673	0,88686
RVD vs. noRVD	BLM hydrolase	0,21525	0,24036	0,80418
RVD vs. noRVD	IL10	0,21341	0,67794	1,00000
RVD vs. noRVD	CD163	0,21327	0,38958	0,93603
RVD vs. noRVD	Flt3L	0,21136	0,22006	0,78726
RVD vs. noRVD	PRSS2	0,21073	0,61048	1,00000
RVD vs. noRVD	ARTN	0,20675	0,26764	0,82358
RVD vs. noRVD	VEGFD	0,20673	0,40997	0,94084
RVD vs. noRVD	U-PAR	0,20669	0,38838	0,93603
RVD vs. noRVD	MEPE	0,20507	0,44651	0,95719
RVD vs. noRVD	CST5	0,20370	0,31609	0,87771
RVD vs. noRVD	TNFRSF13B	0,20368	0,18472	0,72659
RVD vs. noRVD	CD84	0,20072	0,27351	0,82981
RVD vs. noRVD	IL-18R1	0,19802	0,15603	0,67470
RVD vs. noRVD	PARP-1	0,19754	0,56042	1,00000
RVD vs. noRVD	ICAM-2	0,19591	0,33280	0,88253
RVD vs. noRVD	NCAM1	0,19142	0,24406	0,80512
RVD vs. noRVD	TNFRSF14	0,19081	0,42550	0,94146
RVD vs. noRVD	CTSL1	0,18840	0,31627	0,87771
RVD vs. noRVD	PLC	0,18823	0,32561	0,87935
RVD vs. noRVD	CPA1	0,18729	0,70083	1,00000
RVD vs. noRVD	TNFRSF11A	0,18500	0,49718	1,00000
RVD vs. noRVD	TNFSF14	0,18459	0,53200	1,00000
RVD vs. noRVD	CCL15	0,18320	0,40393	0,94084
RVD vs. noRVD	TNF-R1	0,18294	0,55235	1,00000
RVD vs. noRVD	TNFB	0,18066	0,19351	0,73225
RVD vs. noRVD	KLK6	0,17856	0,39545	0,94084
RVD vs. noRVD	LCN2	0,17764	0,30875	0,87771
RVD vs. noRVD	TNFRSF10A	0,17744	0,26570	0,82358
RVD vs. noRVD	PCOLCE	0,17730	0,41913	0,94146
RVD vs. noRVD	PRELP	0,17567	0,02811	0,34238
RVD vs. noRVD	Gal-3	0,17401	0,37147	0,91667
RVD vs. noRVD	ADM	0,17316	0,05172	0,44127
RVD vs. noRVD	IL-4RA	0,17306	0,35117	0,88536
RVD vs. noRVD	IL-17D	0,17080	0,06614	0,45949
RVD vs. noRVD	C2	0,17058	0,20045	0,73225
RVD vs. noRVD	SPARCL1	0,16866	0,19982	0,73225
RVD vs. noRVD	AXL	0,16720	0,47262	0,98192
RVD vs. noRVD	GP1BA	0,16606	0,60803	1,00000

RVD vs. noRVD	NRP1	0,16581	0,11992	0,64079
RVD vs. noRVD	CCL11	0,16549	0,26054	0,82358
RVD vs. noRVD	PCSK9	0,16496	0,34977	0,88536
RVD vs. noRVD	MMP7	0,16253	0,23531	0,80067
RVD vs. noRVD	ACE2	0,16235	0,54731	1,00000
RVD vs. noRVD	TR	0,16162	0,71128	1,00000
RVD vs. noRVD	GAS6	0,16135	0,32914	0,87935
RVD vs. noRVD	IL-17C	0,15907	0,67620	1,00000
RVD vs. noRVD	BOC	0,15882	0,27873	0,83374
RVD vs. noRVD	PLXNB2	0,15875	0,14928	0,67470
RVD vs. noRVD	VEGFA	0,15847	0,40367	0,94084
RVD vs. noRVD	ALCAM	0,15636	0,43932	0,95304
RVD vs. noRVD	PD-L1	0,15629	0,43042	0,94146
RVD vs. noRVD	TIE2	0,15576	0,08507	0,53428
RVD vs. noRVD	CDH5	0,15558	0,50599	1,00000
RVD vs. noRVD	QPCT	0,15512	0,28220	0,83374
RVD vs. noRVD	PAPPA	0,15508	0,46513	0,97952
RVD vs. noRVD	ST1A1	0,15413	0,89950	1,00000
RVD vs. noRVD	SIRT2	0,15385	0,88769	1,00000
RVD vs. noRVD	MET	0,15352	0,07435	0,48394
RVD vs. noRVD	CXCL16	0,15258	0,42826	0,94146
RVD vs. noRVD	AZU1	0,14892	0,73056	1,00000
RVD vs. noRVD	TGF-alpha	0,14807	0,37384	0,91667
RVD vs. noRVD	IL-22 RA1	0,14716	0,62213	1,00000
RVD vs. noRVD	CCL23	0,14547	0,31174	0,87771
RVD vs. noRVD	SELE	0,14438	0,65026	1,00000
RVD vs. noRVD	TIE1	0,14394	0,25655	0,82246
RVD vs. noRVD	HO-1	0,14267	0,16019	0,67470
RVD vs. noRVD	MERTK	0,14109	0,38133	0,92867
RVD vs. noRVD	FGF-5	0,14055	0,32798	0,87935
RVD vs. noRVD	IGFBP6	0,13815	0,56727	1,00000
RVD vs. noRVD	RAGE	0,13761	0,49174	1,00000
RVD vs. noRVD	PAM	0,13715	0,31298	0,87771
RVD vs. noRVD	EGFR	0,13627	0,34569	0,88536
RVD vs. noRVD	PI3	0,13585	0,72075	1,00000
RVD vs. noRVD	IGLC2	0,13544	0,65903	1,00000
RVD vs. noRVD	CD59	0,13387	0,49057	1,00000
RVD vs. noRVD	GRN	0,13377	0,51822	1,00000
RVD vs. noRVD	CEACAM8	0,13070	0,62029	1,00000
RVD vs. noRVD	FABP4	0,12877	0,92284	1,00000
RVD vs. noRVD	LILRB2	0,12831	0,54195	1,00000
RVD vs. noRVD	PRSS27	0,12498	0,56689	1,00000
RVD vs. noRVD	SHPS-1	0,12405	0,62043	1,00000
RVD vs. noRVD	CASP-8	0,12344	0,87598	1,00000
RVD vs. noRVD	SLAMF7	0,12088	0,40775	0,94084
RVD vs. noRVD	PGF	0,12019	0,52810	1,00000
RVD vs. noRVD	PGLYRP1	0,11974	0,73795	1,00000
RVD vs. noRVD	CPB1	0,11890	0,82643	1,00000
RVD vs. noRVD	SOD1	0,11822	0,73491	1,00000
RVD vs. noRVD	CD40-L	0,11773	0,96900	1,00000

RVD vs. noRVD	SAA4	0,11522	0,77263	1,00000
RVD vs. noRVD	VSIG2	0,11406	0,81655	1,00000
RVD vs. noRVD	IFN-gamma	0,11068	0,92061	1,00000
RVD vs. noRVD	ICAM3	0,10990	0,44921	0,95724
RVD vs. noRVD	Gal-4	0,10903	0,73992	1,00000
RVD vs. noRVD	TF	0,10888	0,53801	1,00000
RVD vs. noRVD	LILRB5	0,10881	0,83084	1,00000
RVD vs. noRVD	STAMBP	0,10845	0,93117	1,00000
RVD vs. noRVD	APOM	0,10473	0,65565	1,00000
RVD vs. noRVD	FAP	0,10302	0,23365	0,80067
RVD vs. noRVD	IL4	0,10290	0,40996	0,94084
RVD vs. noRVD	LTBP2	0,10211	0,54762	1,00000
RVD vs. noRVD	MMP-9	0,10090	0,77875	1,00000
RVD vs. noRVD	FS	0,10069	0,68571	1,00000
RVD vs. noRVD	NEMO	0,10015	0,96290	1,00000
RVD vs. noRVD	TCN2	0,09980	0,50953	1,00000
RVD vs. noRVD	PD-L2	0,09899	0,61370	1,00000
RVD vs. noRVD	KIM1	0,09852	0,89870	1,00000
RVD vs. noRVD	CDH1	0,09767	0,74622	1,00000
RVD vs. noRVD	CD93	0,09747	0,72832	1,00000
RVD vs. noRVD	COL1A1	0,09745	0,67283	1,00000
RVD vs. noRVD	GNLY	0,09695	0,67499	1,00000
RVD vs. noRVD	VASN	0,09512	0,44191	0,95304
RVD vs. noRVD	TLT-2	0,09340	0,80283	1,00000
RVD vs. noRVD	LTBR	0,09229	0,77997	1,00000
RVD vs. noRVD	SORT1	0,09110	0,35556	0,88686
RVD vs. noRVD	TNF-R2	0,08775	0,87108	1,00000
RVD vs. noRVD	IL13	0,08646	0,86179	1,00000
RVD vs. noRVD	IL-15RA	0,08564	0,72673	1,00000
RVD vs. noRVD	GDF-2	0,08042	0,88641	1,00000
RVD vs. noRVD	IL-12B	0,08016	0,88602	1,00000
RVD vs. noRVD	FETUB	0,07746	0,85829	1,00000
RVD vs. noRVD	uPA	0,07683	0,58553	1,00000
RVD vs. noRVD	PRSS8	0,07576	0,64666	1,00000
RVD vs. noRVD	ST6GAL1	0,07535	0,78853	1,00000
RVD vs. noRVD	NOTCH1	0,07419	0,65067	1,00000
RVD vs. noRVD	OSM	0,07375	0,90072	1,00000
RVD vs. noRVD	TGFBR3	0,07214	0,78077	1,00000
RVD vs. noRVD	REG3A	0,07181	0,55725	1,00000
RVD vs. noRVD	FCN2	0,07011	0,83967	1,00000
RVD vs. noRVD	LILRB1	0,06974	0,68591	1,00000
RVD vs. noRVD	MCP-2	0,06914	0,89525	1,00000
RVD vs. noRVD	DCN	0,06827	0,64533	1,00000
RVD vs. noRVD	CA5A	0,06646	0,94956	1,00000
RVD vs. noRVD	SELL	0,06494	0,82307	1,00000
RVD vs. noRVD	CR2	0,06299	0,91623	1,00000
RVD vs. noRVD	PTPRS	0,06248	0,67417	1,00000
RVD vs. noRVD	PRCP	0,06247	0,72728	1,00000
RVD vs. noRVD	DEFA1	0,06229	0,88789	1,00000
RVD vs. noRVD	REN	0,06211	0,89944	1,00000

RVD vs. noRVD	CD46	0,06180	0,90960	1,00000
RVD vs. noRVD	PRTN3	0,05642	0,92280	1,00000
RVD vs. noRVD	PLTP	0,05144	0,62198	1,00000
RVD vs. noRVD	NRTN	0,05089	0,93559	1,00000
RVD vs. noRVD	EPHB4	0,05003	0,91871	1,00000
RVD vs. noRVD	IL-18BP	0,04979	0,94108	1,00000
RVD vs. noRVD	MEGF9	0,04948	0,87958	1,00000
RVD vs. noRVD	IL33	0,04853	0,96340	1,00000
RVD vs. noRVD	CRTAC1	0,04762	0,91675	1,00000
RVD vs. noRVD	PROC	0,04756	0,94385	1,00000
RVD vs. noRVD	MCP-3	0,04751	0,94907	1,00000
RVD vs. noRVD	IL2-RA	0,04597	0,95392	1,00000
RVD vs. noRVD	SPON2	0,04499	0,42446	0,94146
RVD vs. noRVD	GLO1	0,04271	0,96581	1,00000
RVD vs. noRVD	TNFRSF9	0,04145	0,95143	1,00000
RVD vs. noRVD	PLA2G7	0,03948	0,89267	1,00000
RVD vs. noRVD	ITGB2	0,03525	0,95502	1,00000
RVD vs. noRVD	BMP-6	0,03455	0,96746	1,00000
RVD vs. noRVD	IL-20	0,03449	0,84903	1,00000
RVD vs. noRVD	CD6	0,03342	0,96311	1,00000
RVD vs. noRVD	CHIT1	0,03321	0,99578	1,00000
RVD vs. noRVD	PSP-D	0,03268	0,98389	1,00000
RVD vs. noRVD	CSTB	0,03197	0,98770	1,00000
RVD vs. noRVD	HB-EGF	0,03117	0,99172	1,00000
RVD vs. noRVD	UMOD	0,03108	0,85765	1,00000
RVD vs. noRVD	FAS	0,02987	0,97162	1,00000
RVD vs. noRVD	MMP-3	0,02944	0,98880	1,00000
RVD vs. noRVD	IL18	0,02767	0,95443	1,00000
RVD vs. noRVD	PIgR	0,02718	0,74504	1,00000
RVD vs. noRVD	IL-6RA	0,02591	0,97902	1,00000
RVD vs. noRVD	CDCP1	0,02528	0,98847	1,00000
RVD vs. noRVD	TSLP	0,02377	0,97567	1,00000
RVD vs. noRVD	MPO	0,02272	0,98394	1,00000
RVD vs. noRVD	LOX-1	0,02173	0,98147	1,00000
RVD vs. noRVD	SLAMF1	0,02063	0,98098	1,00000
RVD vs. noRVD	IL1RL2	0,01912	0,97671	1,00000
RVD vs. noRVD	RETN	0,01758	0,99334	1,00000
RVD vs. noRVD	CX3CL1	0,01701	0,98923	1,00000
RVD vs. noRVD	vWF	0,01534	0,99668	1,00000
RVD vs. noRVD	PTX3	0,01433	0,99270	1,00000
RVD vs. noRVD	IL5	0,01181	0,99683	1,00000
RVD vs. noRVD	TRANCE	0,01135	0,99744	1,00000
RVD vs. noRVD	TM	0,00995	0,99454	1,00000
RVD vs. noRVD	CTSZ	0,00691	0,99817	1,00000
RVD vs. noRVD	ADA	0,00663	0,99806	1,00000
RVD vs. noRVD	XCL1	0,00492	0,99961	1,00000
RVD vs. noRVD	Gal-9	0,00429	0,99856	1,00000
RVD vs. noRVD	TNF	0,00417	0,99919	1,00000
RVD vs. noRVD	LIF	0,00201	0,99905	1,00000
RVD vs. noRVD	CSF-1	0,00054	0,99993	1,00000

RVD vs. noRVD	CD8A	0,00028	1,00000	1,00000
RVD vs. noRVD	Beta-NGF	0,00025	0,43128	0,94146
RVD vs. noRVD	IL2	-0,00012	0,99999	1,00000
RVD vs. noRVD	SERPINA7	-0,00243	0,99977	1,00000
RVD vs. noRVD	FGF-21	-0,00460	0,99986	1,00000
RVD vs. noRVD	CA4	-0,00751	0,99548	1,00000
RVD vs. noRVD	hOSCAR	-0,00951	0,98523	1,00000
RVD vs. noRVD	SOD2	-0,01021	0,91388	1,00000
RVD vs. noRVD	IL-2RB	-0,01665	0,92945	1,00000
RVD vs. noRVD	PON3	-0,01918	0,99364	1,00000
RVD vs. noRVD	CA3	-0,01995	0,98363	1,00000
RVD vs. noRVD	CCL19	-0,02061	0,99298	1,00000
RVD vs. noRVD	THBS2	-0,02224	0,92638	1,00000
RVD vs. noRVD	TNFRSF10C	-0,02490	0,98198	1,00000
RVD vs. noRVD	MBL2	-0,03204	0,99370	1,00000
RVD vs. noRVD	SERPINA5	-0,03281	0,96881	1,00000
RVD vs. noRVD	4E-BP1	-0,03440	0,96656	1,00000
RVD vs. noRVD	ITGAM	-0,03553	0,89236	1,00000
RVD vs. noRVD	IL-10RB	-0,03702	0,91076	1,00000
RVD vs. noRVD	ADAM-TS13	-0,03776	0,51467	1,00000
RVD vs. noRVD	CNDP1	-0,04147	0,95764	1,00000
RVD vs. noRVD	F7	-0,04228	0,93729	1,00000
RVD vs. noRVD	TGM2	-0,04944	0,86786	1,00000
RVD vs. noRVD	MARCO	-0,05107	0,56627	1,00000
RVD vs. noRVD	DLK-1	-0,05151	0,97013	1,00000
RVD vs. noRVD	HSP 27	-0,05335	0,78130	1,00000
RVD vs. noRVD	IL-17RA	-0,05358	0,92754	1,00000
RVD vs. noRVD	MFAP5	-0,05556	0,77881	1,00000
RVD vs. noRVD	MMP-10	-0,05642	0,93819	1,00000
RVD vs. noRVD	F11	-0,06193	0,84471	1,00000
RVD vs. noRVD	Ep-CAM	-0,06778	0,95908	1,00000
RVD vs. noRVD	DNER	-0,07179	0,64933	1,00000
RVD vs. noRVD	LPL	-0,07189	0,74603	1,00000
RVD vs. noRVD	IDUA	-0,07347	0,80604	1,00000
RVD vs. noRVD	FABP2	-0,07491	0,89223	1,00000
RVD vs. noRVD	IL-10RA	-0,07978	0,80780	1,00000
RVD vs. noRVD	IL-17A	-0,08776	0,85145	1,00000
RVD vs. noRVD	PSGL-1	-0,08918	0,47450	0,98192
RVD vs. noRVD	TRAIL	-0,09132	0,55152	1,00000
RVD vs. noRVD	TIMP4	-0,09694	0,84443	1,00000
RVD vs. noRVD	IL-1ra	-0,10399	0,82148	1,00000
RVD vs. noRVD	LDL receptor	-0,10713	0,79517	1,00000
RVD vs. noRVD	IL-20RA	-0,11235	0,78765	1,00000
RVD vs. noRVD	FGF-19	-0,11326	0,83649	1,00000
RVD vs. noRVD	IL16	-0,11562	0,66357	1,00000
RVD vs. noRVD	MMP12	-0,11822	0,80715	1,00000
RVD vs. noRVD	CD5	-0,14910	0,24513	0,80512
RVD vs. noRVD	SCF	-0,16929	0,34700	0,88536
RVD vs. noRVD	AMBP	-0,17939	0,02907	0,34238
RVD vs. noRVD	CES1	-0,19163	0,59783	1,00000

RVD vs. noRVD	MB	-0,20486	0,57348	1,00000
RVD vs. noRVD	IL-24	-0,21110	0,10867	0,60412
RVD vs. noRVD	CFHR5	-0,21146	0,30754	0,87771
RVD vs. noRVD	GIF	-0,22827	0,64096	1,00000
RVD vs. noRVD	TR-AP	-0,24815	0,22434	0,78738
RVD vs. noRVD	EN-RAGE	-0,24949	0,18108	0,72659
RVD vs. noRVD	CHI3L1	-0,25766	0,45637	0,96675
RVD vs. noRVD	LEP	-0,30714	0,47127	0,98192
RVD vs. noRVD	SERPINA12	-0,35510	0,40703	0,94084
RVD vs. noRVD	HAOX1	-0,36412	0,61913	1,00000
