# Supplementary material for: FGF-23 is a biomarker of RV dysfunction and congestion in patients with HFrEF
Source: Sci Rep. 2023 Sep 25;13:16004. doi: 10.1038/s41598-023-42558-4 (PMC10520041; doi:10.1038/s41598-023-42558-4)
Supplement: Supplementary file 5 — Supplementary Table 4. [file 41598_2023_42558_MOESM5_ESM.docx]

**Online supplement Table 4. Parameters associated with FGF-23 level**


eGFR, estimated glomerular filtration rate; Hb1Ac, glycated hemoglobin; LVEF, left ventricular ejection fraction; RV, right ventricular

| **Variable** | **Univariable regression** |  | **Multivariable regression** |
| --- | --- | --- | --- |
|  | **r^2^** | **p** | **p** |
| LVEF *(%)* | 0.014 | **0.03** | 0.44 |
| RV dysfunction grade *(1-4)* | 0.07 | **<0.0001** | **0.004** |
| Subjective congestion *(present vs. absent)* | 0.05 | **<0.0001** | **0.004** |
| eGFR *(ml.min^-1^.1.73m^-2^)* | 0.02 | **0.02** | **0.04** |
| Hb1Ac (mmol/mol) | 0.01 | **0.04** | 0.30 |
